# Supplementary figures and images for: Participation in microfinance based Self Help Groups in India: Who becomes a member and for how long?
Source: PLoS One. 2020 Aug 18;15(8):e0237519. doi: 10.1371/journal.pone.0237519 (PMC7437468; doi:10.1371/journal.pone.0237519)

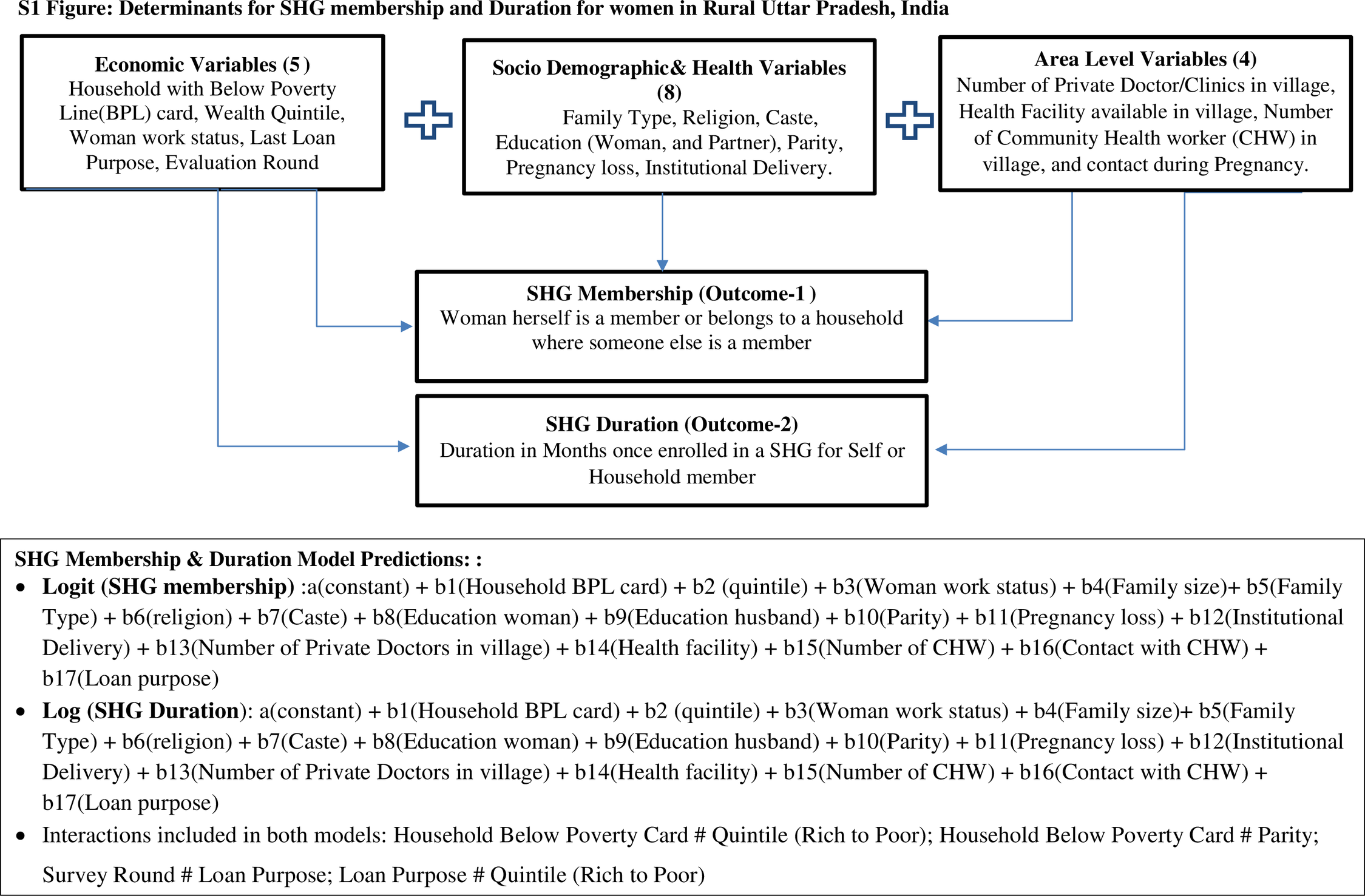

Supplement: S1 Appendix — (TIF) [file pone.0237519.s001.tif]
