# Supplementary material for: Participation in microfinance based Self Help Groups in India: Who becomes a member and for how long?
Source: PLoS One. 2020 Aug 18;15(8):e0237519. doi: 10.1371/journal.pone.0237519 (PMC7437468; doi:10.1371/journal.pone.0237519)
Supplement: S2 Appendix — (DOCX) [file pone.0237519.s002.docx]

**S2 APPENDIX**

**Table: Different count data models for SHG Duration with Goodness of fit measures**

| **Regression Model** | **Number of Observations** | **No of Parameters** | **Log-Likelihood** | **CAIC** | **BIC** |
| --- | --- | --- | --- | --- | --- |
| Poisson | 15,300 | 22 | -267,857 | 535,827 | 536,255 |
| Negative Binomial | 15,300 | 22 | -50,652 | 101,419 | 101,854 |
| Zero Inflated Poisson | 15,300 | 22 | -78,252 | 156,620 | 157,063 |
| Zero Inflated Negative Binomial | 15,300 | 22 | -35,779 | 71,676 | 72,127 |
| Hurdle Negative Binomial | 15,300 | 22 | -45,713 | 91,653 | 92,516 |

**Notes:** CAIC: Consistent Akaike Information Criterion; BIC: Bayesian Information Criterion
