# Supplementary material for: Participation in microfinance based Self Help Groups in India: Who becomes a member and for how long?
Source: PLoS One. 2020 Aug 18;15(8):e0237519. doi: 10.1371/journal.pone.0237519 (PMC7437468; doi:10.1371/journal.pone.0237519)
Supplement: S3 Appendix — (DOCX) [file pone.0237519.s003.docx]

**S3 APPENDIX**

**Table: Summary statistics of selected variables by Non-Self-Help Group [Non-SHG]Household and Self-Help Group [SHG] Households.**

|  | **Variable** | **Summary Statistics(N=15,300)** | |
| --- | --- | --- | --- |
| Sr.no |  | **Non-SHG Households(=0)**  **Mean/Proportion (SD)** | **SHG Households(=1)**  **Mean/Proportion (SD)** |
|  | ***Dependent Variable*** | | |
| 1. | SHG Membership status (=1 if the eligible woman is herself a member or belongs to an SHG member household) | 47% | 53% |
| 2. | SHG Duration (in completed months once enrolled into a SHG) | NA | 42  (33) |
|  | ***Independent Variables: Economic Characteristics*** | | |
| 3. | **Household (HH) with Below Poverty Line (BPL) Card** | 40% | 51% |
| 4. | **Household Wealth Quintile (Poor to Poorest )** |  |  |
|  | - - - 1. Marginally Poor | 22% | 19% |
|  | - - - 1. Moderately Poor | 19% | 20% |
|  | - - - 1. Poor | 20% | 21% |
|  | - - - 1. Poorer | 20% | 20% |
|  | - - - 1. Poorest | 19% | 20% |
| 5. | **Purpose of Last Loan Borrowed by SHG and Non-SHG Members from any source.** |  |  |
|  | 1. No Loan Taken | 72% | 61% |
|  | 1. Enterprise Purposes (To start or expand the business) | 3% | 10% |
|  | 1. Non-Enterprise(Repay old loan, Child’s education or Marriage, House Repairs) | 6% | 11% |
|  | 1. Health & Illness(For Treatment of Illness or Delivery) | 5% | 12% |
|  | 1. .Others(Reason not stated) | 4% | 6% |
|  | ***Independent Variables: Socio-demographic and Health aspects*** | | |
| 6. | **Eligible Woman Presently Working to earn in cash, kind or both** | 13% | 17% |
| 7. | **Joint and Extended Family Household** | 56% | 61% |
| 8. | **Religion** |  |  |
|  | Muslim | 9% | 7% |
|  | Hinduism & Others | 91% | 93% |
| 9. | **Scheduled Caste** |  |  |
|  | General Caste | 14% | 11% |
|  | Other Backward Caste | 44% | 40% |
|  | Scheduled Tribe | 6% | 5% |
|  | Scheduled Caste | 36% | 44% |
| 10 | **Eligible Woman (EW)Age in completed years** | 26  (4.6) | 26  (4.6) |
| 11. | **Eligible Woman(EW’s) Education Level** |  |  |
|  | No schooling | 34% | 33% |
|  | Completed Primary& Middle School(year nine) | 38% | 41% |
|  | Completed Secondary(up to year 10) and Above | 28% | 26% |
| 12. | **EW’s Husband Education Level** |  |  |
|  | No schooling | 17% | 17% |
|  | Completed Primary& Middle School(year nine) | 46% | 51% |
|  | Completed Secondary(year 10) and Above | 37% | 32% |
| 13. | **Parity of Eligible Woman** | 2.3  (1.4) | 2.5  (1.5) |
| 14. | **Total Pregnancy Loss** | 0.35  (0.76) | 0.41  (0.83) |
| 15. | **Place of Last Delivery** |  |  |
|  | Home Delivery | 12% | 9% |
|  | Institutional Delivery | 88% | 91% |
|  | ***Independent Variables: Area ( Village) Level Characteristics*** | | |
| 16. | **Mean Number of Private Doctor Clinics in village** | 0.19  (0.76) | 0.23  (0.96) |
| 17. | **Mean Number of ASHA & ANM in the village** | 1.8  (2.1) | 2.0  (2.4) |
| 18. | **Availability of Any Govt. health facility in the village** |  |  |
|  | No Health Facility available in the village | 28% | 26% |
|  | Only Government Facility in village | 58% | 58% |
|  | Only Private Facility in village | 2% | 2% |
|  | Both Government and Private in the village | 12% | 14% |
| 19. | **Mean number of contact with ASHA/ANM/AWW/SHG in last pregnancy** | 3.7  (5.3) | 4.5  (5.7) |
|  | ***Independent Variables: Round Characteristics*** | | |
| 20. | **Evaluation Survey Round** |  |  |
|  | Round 1 /Baseline-2015 (=0) | 61% | 51% |
|  | Round 2/ Endline-2017 (=1) | 9% | 49% |

**Acronyms:** Eligible woman(EW), Accredited Social Health Worker (ASHA)/Auxiliary Nurse Midwife(ANM) Anganwadi worker(AWW)-ASHA/ANM &AWW are government health workers situated in villages as per population guidelines and provide preventative maternal, child and other health services.
