# Supplementary material for: Participation in microfinance based Self Help Groups in India: Who becomes a member and for how long?
Source: PLoS One. 2020 Aug 18;15(8):e0237519. doi: 10.1371/journal.pone.0237519 (PMC7437468; doi:10.1371/journal.pone.0237519)
Supplement: S4 Appendix — (DOCX) [file pone.0237519.s004.docx]

**S4 APPENDIX**

**Table. Results from the Multivariate Logit Regression for Determinants of SHG Membership and Hurdle Negative Binomial Regression for Determinants of SHG Duration of Membership**

|  |  | **SHG Membership Logit Analysis** | | | | | | **SHG Duration:**  **Hurdle Negative Binomial Analysis** | |
| --- | --- | --- | --- | --- | --- | --- | --- | --- | --- |
| **S.no** | **Variable name** | **Model I (Economic variables only)** | | **Model II (Economic + Socio-demographic variables)** | | **Model III (Economic+ Socio-demographic+ Area Variables)** | | **Model III ( Economic + Socio-demographic + Area Variables)** | |
|  |  | OR (95% CI) | | OR (95% CI) | | OR (95% CI) | | IRR (95% CI) | |
| 1 | **Household (HH) has Below Poverty (BLP) Card** |  | |  | |  | |  | |
|  | No(reference) |  | |  | |  | |  | |
|  | Yes | 1.83 ***  (1.58-2.11) | | 2.10 ***  (1.77 -2.50) | | 1.87 ***  (1.57-2.23) | | 0.99  (0.91-1.07) | |
| 2 | **Quintile (rich to poor)** |  | |  | |  | |  | |
|  | 1. Marginally Poor (reference) |  | |  | |  | |  | |
|  | 1. Moderately Poor | 1.32***  (1.15-1.52) | | 1.29***  (1.11-1.48) | | 1.42***  (1.21-1.67) | | 0.93*  (0.85-1.01) | |
|  | 1. Poor | 1.29***  (1.12-1.48) | | 1.23**  (1.06-1.43) | | 1.35***  (1.14-1.59) | | 0.86***  (0.79-0.93) | |
|  | 1. Poorer | 1.35***  (1.18-1.55) | | 1.32***  (1.14-1.53) | | 1.65***  (1.39-1.94) | | 0.81***  (0.74-0.89) | |
|  | 1. Poorest | 1.35***  (1.18-1.54) | | 1.27***  (1.10-1.48) | | 1.71***  (1.45-2.02) | | 0.78***  (0.71-0.85) | |
| 3. | **HH has Below Poverty Card # Quintile (Rich to Poor)** |  | |  | |  | |  | |
|  | BPL card (Yes)# Quintile 1 (Marginally Poor)(reference) |  | |  | |  | |  | |
|  | BPL(Yes) #Quintile 2  (Moderately Poor) | 0.87  (0.71-1.07) | | 0.86  (0.71-1.06) | | 0.92  (0.74-1.13) | | 1.07  (0.96-1.17) | |
|  | BPL(Yes) # Quintile 3 (Poor) | 0.85  (0.70-1.04) | | 0.85  (0.70-1.05) | | 0.94  (0.76-1.16) | | 1.05  (0.95-1.16) | |
|  | BPL(Yes) # Quintile 4 (Poorer) | 0.73***  (0.59-0.89) | | 0.74***  (0.60-0.91) | | 0.80**  (0.65-1.00) | | 1.04  (0.94-1.15) | |
|  | BPL(Yes )# Quintile 5  ( Poorest) | 0.77***  (0.63-0.94) | | 0.81**  (0.65-1.00) | | 0.89  (0.71-1.11) | | 1.04  (0.94-1.15) | |
| 4 | **Eligible Woman Presently Working status** |  | |  | |  | |  | |
|  | Not Working (reference) |  | |  | |  | |  | |
|  | Presently Working to earn in cash, kind or both | 1.30***  (1.19-1.43) | | 1.25***  (1.14-1.38) | | 1.25***  (1.13-1.38) | | 0.99  (0.94-1.03) | |
| 5 | **Type of Family** |  | |  | |  | |  | |
|  | Nuclear Family (reference) |  | |  | |  | |  | |
|  | Joint & Extended Family |  | | 1.34***  (1.24-1.45) | | 1.33***  (1.23-1.44) | | 1.18***  (1.13-1.22) | |
| 6 | **Religion (Household Head)** |  | |  | |  | |  | |
|  | Muslim Household(reference) |  | |  | |  | |  | |
|  | Hinduism & Others |  | | 1.17***  (1.04-1.33) | | 1.19***  (1.05-1.35) | | 0.96  (0.90-1.02) | |
| 7 | **Caste** |  | |  | |  | |  | |
|  | General Caste(reference) |  | |  | |  | |  | |
|  | Other Backward Caste |  | | 1.04  (0.93-1.15) | | 0.95  (0.85-1.07) | | 0.93***  (0.88-0.99) | |
|  | Scheduled Tribe |  | | 1.02  (0.84-1.18) | | 1.04  (0.87-1.24) | | 0.88***  (0.81-0.97) | |
|  | Scheduled Caste |  | | 1.34***  (1.19-1.50) | | 1.25***  (1.12-1.41) | | 0.86***  (0.81-0.91) | |
| 8 | **Eligible woman (EW) Age in years** |  | | 1.01***  (1.00-1.02) | | 1.01***  (1.00-1.02) | | 1.02***  (1.01-1.02) | |
| 9 | **Eligible woman education level(completed years )** |  | |  | |  | |  | |
|  | No schooling(reference) |  | |  | |  | |  | |
|  | Completed Primary& Middle School( up to year 9) |  | | 1.26***  (1.16-1.37) | | 1.19***  (1.09-1.30) | | 0.97  (0.93-1.02) | |
|  | Completed Secondary(up to year 10) and Above |  | | 1.23***  (1.10-1.36) | | 1.13**  (1.01-1.26) | | 0.91***  (0.86-0.96) | |
| 10 | **EW’s Husband Education level(completed)** |  | |  | |  | |  | |
|  | No schooling(reference) |  | |  | |  | |  | |
|  | Completed Primary& Middle School(up to year 9) |  | | 1.12***  (1.01-1.23) | | 1.09*  (0.98-1.19) | | 1.07***  (1.02-1.11) | |
|  | Completed Secondary(up to year 10) and Above |  | | 0.92  (0.82-1.03) | | 0.93  (0.83-1.05) | | 1.13***  (1.07-1.19) | |
| 11 | **Parity** |  | | 1.12***  (1.08-1.17) | | 1.10***  (1.06-1.15) | | 0.98*  (0.96-1.00) | |
| 12 | **HH Below poverty Card # Parity** |  | |  | |  | |  | |
|  | No BPL card # Parity(reference) |  | |  | |  | |  | |
|  | Yes card# Parity |  | | 0.90***  (0.86-0.95) | | 0.89***  (0.85-0.94) | | 1.01  (0.99-1.03) | |
| 13 | **Total Pregnancy Loss** |  | | 1.09***  (1.04-1.14) | | 1.05**  (1.00-1.09) | | 1.02**  (1.00-1.04) | |
| 15 | **Place of Last Delivery** |  | |  | |  | |  | |
|  | Home Delivery(reference) |  | |  | |  | |  | |
|  | Institutional Delivery |  | |  | | 1.10*  (0.98-1.23) | | 1.09**  (1.01-1.17) | |
| 15 | **Availability of Health facility in the village** |  | |  | |  | |  | |
|  | No Health Facility(reference) |  | |  | |  | |  | |
|  | Only Government Health Facility |  | |  | | 0.95  (0.88-1.03) | | 1.03**  (0.99-1.07) | |
|  | Only Private Health Facility |  | |  | | 0.94  (0.71-1.26) | | 1.03  (0.92-1.16) | |
|  | Both Government & Private Health Facility |  | |  | | 0.83  (0.83-1.11) | | 1.06**  (1.00-1.13) | |
| 16 | **Total Number of Private Doctor Clinics in village** |  | |  | | 1.02  (0.97-1.07) | | 0.99  (0.98-1.01) | |
| 17 | **Number of community health workers(ASHA & ANM) in the village** |  | |  | | 1.06***  (1.04-1.08) | | 0.98***  (0.98-0.99) | |
| **18.** | **Number of contact with ASHA/ANM/AWW/ SHG in last pregnancy** |  | |  | | 1.01***  (1.01-1.02) | | 1.00  (1.00-1.00) | |
| 19. | **Last Loan Purpose** |  | |  | |  | |  | |
|  | No Loan availed(reference) |  | |  | |  | |  | |
|  | Enterprise reasons |  | |  | | 4.52***  (3.08-6.63) | | 1.73***  (1.52-1.97) | |
|  | Non- Enterprise reasons |  | |  | | 3.10***  (2.24-4.28) | | 1.78***  (1.56-2.03) | |
|  | Health and Illness |  | |  | | 1.71***  (1.28-2.27) | | 1.38***  (1.20-1.60) | |
|  | Others(Reason not stated) |  | |  | | 2.28***  (1.43-3.63) | | 1.42***  (1.18-1.71) | |
| 20 | **Evaluation Round** |  | |  | |  | |  | |
|  | Round1(reference) |  | |  | |  | |  | |
|  | Round 2 |  | |  | | 1.63***  (1.48-1.78) | | 2.69***  (2.56-2.82) | |
| 21 | **Round# Last Loan Purpose** |  | |  | |  | |  | |
|  | Round 1#NoLoan (reference) |  | |  | |  | |  | |
|  | Round 2# Loan for Enterprise reasons |  | |  | | 0.42***  (0.30-0.60) | | 0.55***  (0.50-0.61) | |
|  | Round 2#Loan for Non – Enterprise reasons |  | |  | | 0.72***  (0.57-0.94) | | 0.59***  (0.54-0.66) | |
|  | Round 2#Loan for Health and Illness reasons |  | |  | | 0.62***  (0.50-0.76) | | 0.61***  (0.53-0.70) | |
|  | Round 2# Loans for Other purposes |  | |  | | 0.48***  (0.34-0.69) | | 0.61***  (0.53-0.69) | |
| 22 | **Last Loan Purpose# Quintile (Rich to Poor)** |  | |  | |  | |  | |
|  | No Loan Taken # Quintile 1 (reference) |  | |  | |  | |  | |
|  | Loan for Enterprise Reasons#  Quintile 2 (Moderately Poor) |  | |  | | 1.31  (0.82-2.08) | | 1.09  (0.95-1.26) | |
|  | Loan for Non-Enterprise Reasons# Quintile 2(Moderately Poor) |  | |  | | 0.64***  (0.44-0.92) | | 0.98  (0.86-1.12) | |
|  | Loan for Health Reasons#  Quintile 2(Moderately Poor) |  | |  | | 0.56***  (0.40-0.79) | | 1.18**  (1.01-1.37) | |
|  | Loan for Other Reasons#  Quintile 2(Moderately Poor) |  | |  | | 1.18  (0.71-1.97) | | 1.02  (0.86-1.22) | |
|  | Loan for Enterprise Reasons#  Quintile 3(Poor) |  | |  | | 1.31  (0.83-2.08) | | 1.09  (0.94-1.26) | |
|  | Loan for Non-Enterprise Reasons # Quintile3(Poor) |  | |  | | 0.61***  (0.42-0.87) | | 1.11  (0.97-1.26) | |
|  | Loan for Health Reasons # Quintile 3(Poor) |  | |  | | 0.66***  (0.47-0.91) | | 1.13*  (0.98-1.31) | |
|  | **Last Loan Purpose# Quintile (Rich to Poor) continued** |  | |  | |  | |  | |
|  | Loan for Other Reasons# Quintile 3(Poor) |  | |  | | 1.35  (0.82-2.22) | | 1.09  (0.93-1.29) | |
|  | Loan for Enterprise Reasons#  Quintile 4(Poorer) |  | |  | | 1.69**  (1.00-2.85) | | 0.86**  (0.75-0.99) | |
|  | Loan for Non-Enterprise Reasons #Quintile 4(Poorer) |  | |  | | 0.75  (0.49-1.13) | | 1.06  (0.91-1.23) | |
|  | Loan for Health Reasons #  Quintile 4(Poorer) |  | |  | | 0.55***  (0.39-0.76) | | 1.15  (0.99-1.33) | |
|  | Loan for Other Reasons#  Quintile 4(Poorer) |  | |  | | 0.64*  (0.39-1.04) | | 1.12  (0.42-1.34) | |
|  | Loan for Enterprise Reasons#  Quintile 5(Poorest) |  | |  | | 1.52  (0.86-2.69) | | 0.89*  (0.76-1.04) | |
|  | Loan for Non-Enterprise Reasons #Quintile 5(Poorest) |  | |  | | 0.69*  (0.45-1.04) | | 0.98  (0.85-1.14) | |
|  | Loan for Health Reasons#  Quintile 5(Poorest) |  | |  | | 0.44***  (0.31-0.61) | | 1.11  (0.96-1.30) | |
|  | Loan for Other Reasons#  Quintile 5(Poorest) |  | |  | | 0.64*  (0.38-1.07) | | 1.02  (0.85-1.22) | |
|  | **Estimation of Model Fit** | **SHG Membership(Logit)** | | | | | | **SHG Duration(HNB)** | |
|  |  | **Model I** | | **Model II** | | **Model III** | | **Model III** | |
|  | Log likelihood | -10,584 | | -10,331 | | -9,945 | | -45,713 | |
|  | Number of Observation | 15,300 | | 15,300 | | 15,300 | | 15,300 | |
|  | AIC/BIC | 20,942 | 21026 | 20,711 | 20,895 | 20,002 | 20,430 | 91,653 | 92,516 |

**Note**: Confidence intervals in parentheses; and significant p-value showing 0.01***,0.05** and 0.10* levels. Log-likelihood and AIC/BIC values were also reported.
