# Supplementary material for: Participation in microfinance based Self Help Groups in India: Who becomes a member and for how long?
Source: PLoS One. 2020 Aug 18;15(8):e0237519. doi: 10.1371/journal.pone.0237519 (PMC7437468; doi:10.1371/journal.pone.0237519)
Supplement: Attachment — Submitted filename: PONE-D-20-01535_reviewer_Feb 2020_review added.pdf [file pone.0237519.s005.pdf]

PLOS ONE

Participation in Micro-finance based Self Help Groups in India: Who becomes a Member and for How Long

--Manuscript Draft--

**Manuscript Number:**	PONE-D-20-01535
**Article Type:**	Research Article
**Full Title:**	Participation in Micro-finance based Self Help Groups in India: Who becomes a Member and for How Long
**Short Title:**	Determinants of Self Help Group Membership and Duration In Rural India
**Corresponding Author:**	Danish Ahmad, MBBS,MSc<br>University of Canberra Faculty of Business Government and Law<br>Canberra, AUSTRALIA
**Keywords:**	Membership; Duration; Participation; Determinants; Community programs; Credit or Saving based programs; Women Empowerment; Uttar Pradesh, India
**Abstract:**	Microfinance is a widely promoted developmental initiative to provide poor women with affordable financial services for poverty alleviation. One adaption of microfinance widespread in South Asia is the Self-Help Group (SHG), Model. India adopted this model in 2011 as part of a federal poverty alleviation program and as a secondary approach to integrating health literacy services for rural women. However, the evidence is limited on who joins such programs and how long they stay SHG members. This paper examines the determinants of membership and duration of membership (outcomes) in an integrated microfinance and health literacy program from one of India's poorest and the most populated states, Uttar Pradesh. This study examines determinants across a range of explanatory variables related to economic, sociodemographic and area-level characteristics. Method: Using secondary survey data from the Uttar Pradesh Community Mobilization project comprising 15,300 women from SHGs and Non-SHG households in rural India, we performed multivariate logistic and hurdle negative binomial regression analyses to model SHG membership and duration. Results: While in general poor women are more likely to be SHG members based on an income threshold limit (the Government-sponsored BPL cards), however, when the poor women are further classified based on asset-based wealth quintiles it appears women from the lowest wealth quintile (poorest group) are less likely to become members but are more likely to become members only when they borrowed for health reasons. On the contrary, women from the two lowest wealth quintiles are more likely to continue longer as members if they borrowed for enterprise or income-generating activities and not for health expenses<br>The study also finds that increasing number of previous pregnancies (parity) is associated with a higher likelihood of membership in contrasts with another study from India which reported a negative associated of higher parity with membership Also, the odds of membership are associated with higher number of pregnancy loss(es) and higher contact with community health workers. Conclusion: This paper generates evidence on who becomes a member of these community-based microfinance programs and for how long. Based on the findings,the study recommends that microfinance programs need to examine their inclusion and retention strategies in favour of the poorest households using multi dimensional indicators that are better able to capture poverty in its myriad forms. The study also shows that the addition of a maternal health literacy component is found to strengthen the underlying microfinance platform as members gain added value from membership.
**Order of Authors:**	Danish Ahmad, MBBS,MSc
	Itismita Mohanty
	Laili Irani
	Dileep Mavalankar
	Theo Niyonseng
**Additional Information:**	

Powered by Editorial Manager® and ProduXion Manager® from Aries Systems Corporation

Question	Response
**Financial Disclosure**<br><br>Enter a financial disclosure statement that describes the sources of funding for the work included in this submission. Review the submission guidelines for detailed requirements. View published research articles from *PLOS ONE* for specific examples.<br><br>This statement is required for submission and **will appear in the published article** if the submission is accepted. Please make sure it is accurate.<br><br>**Unfunded studies**<br>Enter: *The author(s) received no specific funding for this work.*<br><br>**Funded studies**<br>Enter a statement with the following details:<br>• Initials of the authors who received each award<br>• Grant numbers awarded to each author<br>• The full name of each funder<br>• URL of each funder website<br>• Did the sponsors or funders play any role in the study design, data collection and analysis, decision to publish, or preparation of the manuscript?<br>• **NO** - Include this sentence at the end of your statement: *The funders had no role in study design, data collection and analysis, decision to publish, or preparation of the manuscript.*<br>• **YES** - Specify the role(s) played.<br><br>* typeset	The paper is part of the PhD research of the first author(Danish Ahmad) who is supported by the University of Canberra's Higher Degree by Research scholarship for his PhD. Open access funding for the publication is provided by the Bill and Melinda Gates Foundation who also funded the program evaluated in the paper. The funders had no role in study design, data collection and analysis, decision to publish, or preparation of the manuscript.
**Competing Interests**<br><br>Use the instructions below to enter a competing interest statement for this submission. On behalf of all authors, disclose any competing interests that could be perceived to bias this work—acknowledging all financial support and any other relevant financial or non-financial competing interests.<br><br>This statement **will appear in the published article** if the submission is	The authors have declared that no competing interests exist.

accepted. Please make sure it is accurate. View published research articles from *PLOS ONE* for specific examples.<br><br>**NO authors have competing interests**<br><br>Enter: *The authors have declared that no competing interests exist.*<br><br>**Authors with competing interests**<br><br>Enter competing interest details beginning with this statement:<br><br>*I have read the journal's policy and the authors of this manuscript have the following competing interests: [insert competing interests here]*<br><br>* typeset	
**Ethics Statement**<br><br>Enter an ethics statement for this submission. This statement is required if the study involved:<br><br>• Human participants<br>• Human specimens or tissue<br>• Vertebrate animals or cephalopods<br>• Vertebrate embryos or tissues<br>• Field research<br><br>Write "N/A" if the submission does not require an ethics statement.<br><br>General guidance is provided below. Consult the submission guidelines for detailed instructions. **Make sure that all information entered here is included in the Methods section of the manuscript.**	Ethics approval was granted by the Human Research Ethics Committee, at the University of Canberra. Previously, ethics approval for the primary data collection under the IMFHL project was approved by the Independent Ethics Committee of Population Council, New York

**Format for specific study types**<br><br>**Human Subject Research (involving human participants and/or tissue)**<br>• Give the name of the institutional review board or ethics committee that approved the study<br>• Include the approval number and/or a statement indicating approval of this research<br>• Indicate the form of consent obtained (written/oral) or the reason that consent was not obtained (e.g. the data were analyzed anonymously)<br><br>**Animal Research (involving vertebrate animals, embryos or tissues)**<br>• Provide the name of the Institutional Animal Care and Use Committee (IACUC) or other relevant ethics board that reviewed the study protocol, and indicate whether they approved this research or granted a formal waiver of ethical approval<br>• Include an approval number if one was obtained<br>• If the study involved *non-human primates*, add *additional details* about animal welfare and steps taken to ameliorate suffering<br>• If anesthesia, euthanasia, or any kind of animal sacrifice is part of the study, include briefly which substances and/or methods were applied<br><br>**Field Research**<br><br>Include the following details if this study involves the collection of plant, animal, or other materials from a natural setting:<br>• Field permit number<br>• Name of the institution or relevant body that granted permission	
**Data Availability**<br><br>Authors are required to make all data underlying the findings described fully available, without restriction, and from the time of publication. PLOS allows rare exceptions to address legal and ethical concerns. See the PLOS Data Policy and FAQ for detailed information.	No - some restrictions will apply

Powered by Editorial Manager® and ProduXion Manager® from Aries Systems Corporation

A Data Availability Statement describing where the data can be found is required at submission. Your answers to this question constitute the Data Availability Statement and **will be published in the article**, if accepted.<br><br>**Important:** Stating 'data available on request from the author' is not sufficient. If your data are only available upon request, select 'No' for the first question and explain your exceptional situation in the text box.<br><br>Do the authors confirm that all data underlying the findings described in their manuscript are fully available without restriction?	
**Describe where the data may be found in full sentences. If you are copying our sample text, replace any instances of XXX with the appropriate details.**<br><br>• If the data are **held or will be held in a public repository**, include URLs, accession numbers or DOIs. If this information will only be available after acceptance, indicate this by ticking the box below. For example: *All XXX files are available from the XXX database (accession number(s) XXX, XXX.).*<br>• If the data are all contained **within the manuscript and/or Supporting Information files**, enter the following: *All relevant data are within the manuscript and its Supporting Information files.*<br>• If neither of these applies but you are able to provide **details of access elsewhere**, with or without limitations, please do so. For example:<br><br>*Data cannot be shared publicly because of [XXX]. Data are available from the XXX Institutional Data Access / Ethics Committee (contact via XXX) for researchers who meet the criteria for access to confidential data.*<br><br>*The data underlying the results presented in the study are available from (include the name of the third party*	Data will be available upon request

and contact information or URL).<br>• This text is appropriate if the data are owned by a third party and authors do not have permission to share the data.<br><br>* typeset	
Additional data availability information:	Tick here if the URLs/accession numbers/DOIs will be available only after acceptance of the manuscript for publication so that we can ensure their inclusion before publication.

Powered by Editorial Manager® and ProduXion Manager® from Aries Systems Corporation

manuscript
Click here to access/download;Manuscript;Main Body.docx

Participation in Microfinance based Self Help Groups in India: Who becomes a Member and for How Long

Danish Ahmad[1, 4 *],Itismita Mohanty[2,],Laili Irani[3],Dileep Mavalankar[4],Theo Niyonseng[2]

Author Affiliation:

1. Health Research Institute, Faculty of Health, University of Canberra, Canberra, Australia
2. Health Research Institute, Faculty of Health, University of Canberra, Canberra, Australia
3. Population Council, New Delhi, India
4. Indian Institute of Public Health Gandhinagar(IIPHG),Gujarat, India

***Corresponding Author**
E-mail:Danish.ahmad@canberra.edu.au(DA)

Acknowledgements

We wish to thank Professor Rachel Davey, Health Research Institute(HRI), the University of Canberra for her overall guidance and patiently reviewing the first draft of this paper. The authors would also like to thank the University of Canberra, the Public Health Foundation of India, and Population Council for supporting inter-organisational research collaboration. Lastly, the authors thank the Bill and Melinda Gates Foundation for supporting the dissemination of this research

Abstract:

Microfinance is a widely promoted developmental initiative to provide poor women with affordable financial services for poverty alleviation. One popular adaption in South Asia is the Self-Help Group (SHG) model that India adopted in 2011 as part of a federal poverty alleviation program and as a secondary approach to integrating health literacy services for rural women. However, evidence is limited on who joins and continues in SHG programs. This paper examines the determinants of membership and staying members (outcomes) in integrated microfinance and health literacy program from one of India's poorest and most populated states, Uttar Pradesh across a range of explanatory variables related to economic, sociodemographic and area-level characteristics. **Method:** Using secondary survey data from the Uttar Pradesh Community Mobilization project comprising 15,300 women from SHGs and Non-SHG households in rural India, we performed multivariate logistic and hurdle negative binomial regression analyses to model SHG membership and duration. **Results:** While in *general* poor women are more likely to be SHG members based on an income threshold limit (Government-sponsored BPL cards), however, when poor women are further classified based on asset-based wealth quintiles it appears women from the lowest wealth quintile (poorest group) are less likely to become members, but more likely to become members when they borrowed for health reasons only. Conversely, women from the two lowest wealth quintiles are more likely to continue longer as members if they borrowed for enterprise or income-generating activities and not for health expenses. The study also finds that increasing number of previous pregnancies (parity) is associated with a higher membership likelihood in contrast with another study from India reporting a negative association. **Conclusion:** Microfinance programs need to examine their inclusion and retention strategies in favour of poorest household using multidimensional indicators that are better able to capture poverty in its myriad forms. **Word Count (300)**

Introduction

Microfinance (MF) is widely promoted as a developmental initiative to provide low-income populations with affordable financial services. Reflecting a gradual shift in perception that considered the poor to be previously ‘un-bankable’, microfinance is now an established sector with an investment portfolio over USD 114 billion and a reach of more than 139 million clients across 60 countries (1,2). MF clients are likely to be marginalised rural poor populations, with minimal or no landholding, low levels of literacy, and/or other groups without a bank account; which in 2017, still comprised 1.67 billion people or 22% of the global population (2,3). This is relevant to populations in South Asia, where the highest number of the world’s severely poor live and where more than 60% of global microfinance borrowers are also found, the majority being women (2,3).

While still widely promoted by global institutions as a poverty alleviation *approach*, unregulated microfinance practice in the past has harmed clients by promoting ‘over-indebtedness’ as a result of repeated loan taking with negative implications for livelihoods(4,5). Therefore, there needs to be a renewed focus on what microfinance intends to achieve in terms of social development and what hurdles are in its progress. A 2012 systematic review of microfinance’s impact in Sub Saharan Africa, for example, reviewed 15 studies and found that ‘in some cases, microfinance increased poverty, reduced levels of children’s education and disempowered women’ (4). Some criticism of microfinance found that ‘microfinance is largely *antagonistic* to sustainable economic and social development, and consequently to sustainable poverty reduction’ (5,6).

These views, however, need to be placed in the broader debate and assumptions of what qualifies as a successful MF program. Another aspect that intensifies the debate is that specific MF experiences from different regions, like different community programs, are limited in their

scope of generalizability, as program successes and failures from one region to another may not be comparable given regional and cultural dissimilarities (7,8). The layout of the paper is as follows; the first section provides context on current microfinance evidence with reference to a specialised microfinance model involving women only Self-Help Groups(SHG) popular in South Asia especially India. The next section describes the relative strength of SHGs in capturing poorer population in its membership and challenges associated with identifying various gradients of the poor. The research gaps in determinants of microfinance membership and duration of membership are identified that this study seeks to address. By reviewing membership profiles especially among gradients of the poor and the poorest, the study evaluates available microfinance data from rural India to critically review who benefits from groups and what drives women in rural India to join and stay members in women-only groups.

Microfinance: Evidence so far

This study shifts one step backward in examining the determinants of SHGs membership duration in India, a specialised microfinance model involving women only Self-Help Groups in rural India. The SHG model is popular in South Asia(9). India adopted the SHG program as part of its national poverty alleviation program and is home to a substantial, large poor population.

Self-help Group (SHG): Basic Context

The SHG model in India evolved from early microfinance experiences of the Grameen Bank in Bangladesh, and developmental program in South India in the early 1970-80s (3,11). These formative schemes showed the benefits of providing small loans to disadvantaged women, who then reported higher loan repayment rates but also higher utilisation of loans towards household wellbeing (3,4,13). At its core, the SHG model comprises 10-15 poor adult women (clients)

organised into groups with members mostly from a similar background or socio-economic status (2,11). Targeting clients for inclusion into SHGs is mostly done by governments and Non-Governmental Organizations (NGO), often through a local community facilitator who seeks to identify the poorest households with community assistance and use of locally adapted but objective criteria defining poverty (10,11). SHGs specifically seek to target income poor women, especially the poorest amongst them, who sit at the bottom of the poverty pyramid, where higher socio-economic inequalities and deprivations cluster (2,3,10). Member selection targeting the poor, hence, is a crucial consideration for the success of such programs.

For SHG group to function at a minimum, adult women representing households, are required to broadly follow certain principles(regular meetings -up to four times /month, regular savings, internal lending, regular repayment and book/record keeping (10). In India, rural public banks mainly use these indicators to grade SHG functioning after a new group is formed over three to six months to assess their suitability to receive loans, and the amount of bank loan or credit that can be dispensed (10). Rural banks typically offer SHGs incrementally higher loan amounts or credit limits depending on how well the group continues to perform and return previously borrowed loans (11) which is a collective responsibility of group members. SHG membership are thought to foster a sense of group solidarity and cohesion in supporting each other to repay loans and also a broader ‘peer network’ for women(12,13,14). Therefore, member characteristics are vital in determining not only how well SHGs function but also for how long they continue to function. The available evidence on membership determinants comes from a few studies from rural India that mainly find that while members are poor, they tend to be at or just above poverty thresholds; the poorest are often excluded from membership (14,15).

However, limited studies have reported factors that determine SHG membership and none at the time of this research have explored factors which determine the duration of membership once enrolled in groups.

The few studies (13–16) from India and Nigeria (17) that examined determinants of SHG membership found that membership is positively associated with increasing age for women, explained by greater social mobility afforded to women in traditional settings as they transition to roles of carers within families. While these studies offer guidance, the generalization of these findings is limited due to small sample size (< 300 women) in most of them and the inclusion of a few economic determinants in the analyses. Moreover, other studies from regional India have not adequately described sampling and statistical techniques involved (18–20).

Ensuring social programs and microfinance alike reach the intended populations is an important factor in determining program success. Understanding beneficiary characteristics is also important considering that SHGs are considered a platform that can be a scalable model on which other socioeconomic and health programs can be based. SHGs for example in India are co-opted in village health promotion events such as the Village health Nutrition Day(VHND) where preventive health services are delivered and monitored by SHGs (21). Moreover, studies also show that for many poor populations such as SHG clients, out of pocket health expenses remain a major factor in over-indebtedness and in diverting loans away from enterprise or income-generating activities (10,20,22).

In India, the state of Uttar Pradesh (UP), the most populous with high levels of poverty, low development and health indicators, presents the optimal setting for generating evidence on the reach of microfinance program and participating clients (23, 24). This study examined the determinants of SHG membership and duration of membership once enrolled in integrated microfinance and health literacy program in rural Uttar Pradesh, India. The current study builds

on past studies (14,15,25) that used limited individual and economic variables and investigates using a range of economic, sociodemographic and area-level characteristics exhibiting broader interconnectedness and potentially stronger influence on membership and duration. The findings are intended to enhance our understanding of SHG programs in India and generate evidence to improve program design that may be more generalisable.

Methods

This study used secondary data from an evaluation of an Integrated Microfinance and Health Literacy (IMFHL) program that was implemented from 2012 to 2017 in rural Uttar Pradesh (UP). The IMFHL program, a five-year initiative, sought to reach poor women in villages across UP with a package of microfinance (SHG) services bundled with maternal health and neonatal health literacy components designed to address community-related barriers in adopting preventive health behaviours to reduce maternal and neonatal mortality(25).

Project Context:

The IMFHL program was funded by the Bill and Melinda Gates Foundation (BMGF) and involved a consortium of partners comprising of Public Health Foundation of India (PHFI), Population Council (PC), Boston University (BU), and Rajiv Gandhi Mahila Vikas Pariyojan (RGMVP).

As a first step, SHGs were established and later a maternal and neonatal health literacy intervention was added on. The health literacy component targeted SHG members and their households, primarily, pregnant mothers and mothers who had a baby in the last 12 months. These women primarily received health literacy through a SHG member trained in the health component that comprised of health messages and newborn care infection control and thermal

care skills. Overall, the program used a Behaviour Change Communication (BCC) strategy to disseminate health literacy through various strategies and mediums such as inviting pregnant and new mothers to SHG meetings, targeted health letters to pregnant and new mothers, house visits and exposure to community health video shows developed by the program (25).

Survey Sampling Approach and Study Population

Sampling Procedure

The data was collected from eligible women using a structured questionnaire in two cross-sectional survey waves, 2013 and 2015, from 20 districts(out of a total of 80 districts in U.P state). U.P. is administratively subdivided into 80 districts, 822 blocks and 98,000 Gram panchayats (GP) with a total population of almost 200 million (26).GPs are the smallest administrative units within blocks where SHGs are established. A GP is a larger main village and may have smaller villages attached to it. While the IMFHL program was implemented in GPs across 100 blocks in 33 districts, the survey data were collected from 70 blocks in 20 districts and the sample is representative of the program's coverage area.

The representative survey used a three-stage sampling approach for selecting blocks, gram panchayats (GPs), and finally, households based on the states' administrative hierarchy (25).

The survey collected data from the eligible woman in both IMFHL program and non-program households within the same GPs(villages) following simple random sampling. These non-program households form the comparison group for the analysis of determinants of SGH membership and duration. As SHG programs enrol one member from each household only, each individual woman in the survey represents a household. If more than one eligible woman was found in a household, a random procedure was used to select the eligible respondent. In both survey rounds, data was collected from the same districts, blocks, and GPs, but *not* the

same households or women. Moreover, data was also collected from households in the main GPs and from villages within the main GPs.

Sample Size

The survey comprised a total of 15,300 eligible women among whom 8,048(53%) were SHG members. Data were collected at the individual, household and village levels using separate questionnaires. The eligible respondents comprised currently married women aged 15 to 49 years who had babies in the 12 months preceding the survey; household head; and village representatives (25). In order to capture the individual, households and community/area level of influence on SHG membership and duration, this analysis merged individual-level, household and village level sub-datasets across rounds.

Statistical Analysis

This study examined the determinants of SHG membership and duration of membership for women in rural Uttar Pradesh. The explanatory variables included economic, sociodemographic, health and area-level characteristics. All analyses were performed using Stata 14 (Statacorp, USA).

The two dependent variables, a SHG member or not and duration of membership, are defined as follows. Membership is a binary variable defined and coded as zero (No) for those eligible women that are non-member, and 1 (yes) for a member when the eligible woman is *herself* a member of the SHG or *belongs* to a household where someone else (e.g., mother, mother-in-law, sister in law) is an SHG member. The second outcome, duration of membership for SHG members only, is a count variable defined as the number of completed months of membership in the SHG once enrolled.

The explanatory variables used in this analysis are broadly supported by the program participation and maternal and child health literature,and presented in the conceptual framework (fig 1). The variables broadly represent four levels of potential influence namely, i) economic, ii) sociodemographic and health and iii) area level plus program characteristics.

Figure 1: Analysis framework: Determinants for SHG membership and Duration for women in Rural Uttar Pradesh, India

Economic variables included household poverty defined by having: i)Below Poverty Line (BPL) card, an objective income-based economic indicator of a household's poverty level determined by the Indian Government with eligible poor households provided a BPL card (28, 36), ii)working status of eligible woman for cash, kind or both and iii)constructed wealth quintile using surveyed household's asset ownership. The extent of poverty is not homogenous among the poor with levels or gradations of poverty, extending from the marginally poor to the "poorest" often described to be sitting at the bottom of the poverty pyramid. Principal component analysis (PCA) was used in this paper to construct the wealth score to draw out poverty gradients using household assets and amenities variables. Wealth scores were then categorised into quintile groups comprising an equal proportion of households (20 per cent) and extending from the marginally poor in the first quintile down to the poorest in the fifth quintile. The analysis included additional sociodemographic and health variables namely: family type (nuclear vs. joint), religion (Muslim, Hinduism & Others), household social caste (General Hindu Caste, Other Backward Caste, Scheduled Tribes-protected indigenous population, and Scheduled Caste-a subdivision of the Hindu Caste system which is also a proxy for social marginalization), education level of eligible woman and her husband, parity, place of last delivery, pregnancy loss and number of contacts with health worker in last pregnancy. Area

level variables included: availability of government or private health facility in the village, number of private doctor clinics or pharmacies in a village, number of community health workers in a village. Finally, SHG program-level variables included are loan purpose, defined as borrowing any loan amount of money of more than Rs 500 (USD 7) in the last 3 years preceding the surveys (2015-17) from any source, 'round' that represents either survey round 1 (2015) or 2(2017) and also the interaction of quintile# loan purpose to examine variations in borrowing purposes amongst gradients of the poor that may have different associations with membership and duration.

Separate multivariable regression analyses were conducted for each of the two outcomes. Firstly, to assess the determinants of SHG membership, three forward logit regression models were performed, where explanatory variables were included in groups in a forward selection of stepwise regression. The models assessed the impact of explanatory economic variables first, followed by the inclusion of sociodemographic and health variables in the second and finally, the area-level variables in the third model. The results from these three multivariable logistic regression models are presented in table 3.

Is this necessary, makes it very confusing for reader, without much added value

Secondly, the analyses also investigated the determinants of duration of SHG membership, using a negative binomial hurdle regression model which accommodates for two decisions involved in microfinance membership: first, the decision to join a microfinance group, and then the decision to continue membership contingent on joining (29). More specifically, the model allows for the decision to join a SHG group to be treated independently from the decision to continue membership (duration) in *one integrated model*. The negative binomial hurdle model (29–32) is appropriate for count outcomes with underlying data overdispersion as observed in this data. The Hurdle addresses the excess zeroes found in this data representing non-members

as well as members with less than one-month membership duration, both coded as zero (duration = 0). Functionally, the Hurdle partitions the model into two processes. First, a binary process that generates positive counts for SHG members (‘1’) versus non-member (‘0’) is fit using a Logit model (29). Second, the model processes the positive counts only, i.e. SHG members’ duration using a negative binomial regression model (29). The final model produces a two-component regression output: a binary component that captures the probability of being a member (1) or non-member (0) using Odds ratios (OR), and a count component that predicts the duration of the membership using Incidence Rate Ratio (IRR) (29). Only the IRR is reported in this paper that includes all three groups of explanatory variables in the analysis.

Results from a descriptive analysis of the duration of membership show that the sample variance (31) is much greater than the mean value (21) which is an indication of over-dispersion in the data. In such instances, the negative binomial regression procedure, which is designed to handle such extra Poisson variations would be more appropriate as it produces more valid estimates and best model fit(29). Further descriptive statistics of the dataset show that out of the total 15,300 sampled women, 47 per cent or 7,252 belonged to SHGs (members), and the remaining 53 per cent or 8,048 belonged to Non-SHGs (non-members). Additionally, out of the 8,048 SHG members, 99.5 per cent or 8,003had a membership duration of more than 1 month. Only 0.5 per cent of SHG members or **45** respondents had been members for less than 1 month also coded as zero. Differentiating these zeroes(for members with<1-month membership) from non- members also coded as zeroes are possible through the hurdle process described above.

The suitability of various count data models for the SHG membership duration was further evaluated using the Consistent Akaike Information Criteria (CAIC) and Bayesian Information Criteria (BIC), two valid measures of model fit, parsimony and model selection (29). As shown

in Table 1, the Hurdle negative binomial (HNB) provides one of the lowest CAIC and BIC statistic indicating an appropriate model for this analysis (29).

Table 1. Different count data models for SHG Duration with Goodness of fit measures

Regression Model	Number of Observations	No of Parameters	Log-Likelihood	CAIC	BIC
Poisson	15,300	22	-267,140	534,391	534,819
Negative Binomial	15,300	22	-50,643	101,400	101,835
Zero Inflated Poisson	15,300	22	- 78,037	156,191	156,634
Zero Inflated Negative Binomial	15,300	22	-35,762	71,642	72,092
Hurdle Negative Binomial	15,300	22	-43,963	88,101	88,762

Notes: CAIC: Consistent Akaike Information Criterion; BIC: Bayesian Information Criterion.

The results from the Hurdle Negative Binomial model analysis are also presented in Table 3 (Column 4).

Summary Statistics

Some info on what % of households are part of SHGs in the 20 districts you sampled would be useful. This will give an understanding of the spread of SHGs in the community

Table 2 shows summary statistics for all variables categorized across SHG and Non-SHG household. The statistics reveal that 51 per cent of SHG households had a Below Poverty line (BPL) card(an income-based limit to identify poverty) while 40 per cent of non-members held a BPL card. All SHG member households are not with BPL cards. The descriptive statistics also show comparable characteristics across SHG and non-SHG households with respect to woman's age (mean 26 years), woman education (34 per cent had no schooling), and maternal health indicators. Eligible women across both groups (SHG & Non-SHG) reported a mean parity close to 2.3 reflecting near current Indian fertility rates (median 2.2, range 2.1-4) with close to 90 per cent reporting institutional deliveries (33). SHG members had slightly more contacts (4.5) with frontline health workers during the last pregnancy as compared to non-SHG members (3.7). The program-level variables(purpose of the last loan) statistics show that overall

more SHG member households as compared to non-members borrowed a loan in the last three years which was more than USD 7(RS 500). Moreover, a higher per cent of SHG households compared to non-SHG households borrowed their last loan either for enterprise purpose(to start/expand a business), non-enterprise reason(repay an old loan, child's education or Marriage, house Repairs). However, a larger number of non-SHG (15%) households as compared to SHG (13%) members borrowed their last loans to meet health-related expenses.

15% not much higher than 13%

Table has some errors, have highlighted these, also may be useful to just have descriptives of independent variables

Table 2. Summary statistics of variables by Non-Self-Help Group and Self-Help Group (SHG) Households.

Sr. no	Variable	Summary Statistics(N=15,300)	
	**Dependent Variable**	**Non-SHG Households(=0)**	**SHG Households(=1)**
1	SHG Membership status(=1 if the eligible woman is herself a member or belongs to SHG member household)	7,252 (47%)	8,048 (53%)
2	SHG Duration (in completed months once enrolled in a SHG)	7,252 Mean=0(SD=0)	8,048 Mean =42 (SD = 33)
	**Independent Variable**	**Non-SHG Households**	**SHG Households**
	Economic Characteristics		
3.	**Household (HH) with Below Poverty Line (BPL) Card**	2,896 (40%)	4,075 (51%)
4.	**Household Wealth Quintile(Poor to Poorest)**	7,252	8,048
	1. Marginally Poor	1,506 (21%)	1,601 (20%)
	2. Moderately Poor	1,523 (21%)	1,681 (21%)
	3. Poor	1,375 (19%)	1,718 (21%)
	4. Poorer	1,400 (19%)	1,590 (20%)
	5. Poorest	1,448 (20%)	1,458 (18%)
	Sociodemographic and Health aspects		
5.	**Eligible Woman Presently Working to earn in cash, kind or both**	955 (13%)	1,345 (17%)
6.	**Joint and Extended Family Household**	4,050 (56%)	4,878(61%)

	Variable	Summary Statistics(N=15,300)	
	**Dependent Variable** Correct these	**Non-SHG Households**	**Dependent Variable** Correct these
7.	**Religion**		
	Muslim	675 (9%)	598(7%)
	Hinduism & Others	6,577 (91%)	7,450 (93%)
8.	**Scheduled Caste**		
	General Hindu Caste	1,001 (14%)	887 (11%)
	Other Backward Caste	3,238 (44%)	3,256 (40%)
	Scheduled Tribe	425 (6%)	402 (5%)
	Scheduled Caste	2,588 (36%)	3,503 (44%)
9.	**Eligible Woman (EW)Age in completed years**	Mean = 26 (SD = 4.6)	Mean = 26 (SD = 4.6)
10.	**Eligible Woman (EW) Education Level-Completed**		
	No schooling	2,497 (34%)	2,645(33%)
	Completed Primary& Middle School	2,717 (38%)	3,332(41%)
	Completed Secondaryand Above	2,038 (28%)	2,071(26%)
11.	**EW's Husband Education Level**		
	No schooling	1,274 (17%)	1,346(17%)
	Completed Primary& Middle School	3,329 (46%)	4,119(51%)
	Completed Secondaryand Above	2,649 (37%)	2,583(32%)
12.	**Parity of Eligible Woman**	Mean = 2.3 (SD = 1.4)	Mean=2.5 (SD=1.5)
13.	**Total Pregnancy Loss**	Mean=0.35 (SD=0.76)	Mean=0.41 (SD=0.83)
14.	**Place of Last Delivery**		
	Home Delivery	891 (12%)	761(9%)
	Institutional Delivery	6,361 (88%)	7,287 (91%)
	Area(Village) Level Characteristics		
15.	**Mean Number of Private Doctor Clinics in village**	Mean=0.19 (SD=0.76)	Mean=0.23 (SD=0.96)
16.	**Mean Number of ASHA & ANM in village**	Mean=1.8 (SD=2.1)	Mean=2.0 (SD=2.4)
17.	**Availability of Any Govt. health facility in the village**		
	No Health Facility available in village	2,015 (28%)	2,128 (26%)
	Only Government Facility in village	4,234 (58%)	4,662 (58%)
	Only Private Facility in village	105 (2%)	122 (2%)
	Both Government and Private in village	898 (12%)	1,136 (14%)

Is the mean age the norm for all SHGs, or is this result of your sampling?

	Variable	Summary Statistics(N=15,300)	
	**Dependent Variable**	**Non-SHG Households**	**Dependent Variable**
18.	**Mean Number of contact with ASHA/ANM/AWW/SHG in last pregnancy**	Mean=3.7 (SD=5.3)	Mean=4.5 (SD=5.7)
	Program Level Characteristics		
19.	**Evaluation Survey Round**		
	Round 1 /Baseline-2013 (=0)	4,342 (61%)	3,888 (51%)
	Round 2/ Endline-2015 (=1)	2,745 (39%)	3,804 (49%)
20.	**Purpose of Last Loan Borrowed by SHG and Non-SHG Members from any source.**		
	No Loan Taken	5,203 (72%)	4,909 (61%)
	Enterprise Purposes(To start or expand business)	206 (3%)	772 (10%)
	Non-Enterprise(Repay old loan, Child's education or Marriage, House Repairs)	449 (6%)	924 (11%)
	Health Purpose(For Treatment of Illness or Delivery)	1,092 (15%)	986 (12%)
	Others(reason not stated)	302(4%)	457(6%)

Acronyms: Eligible woman(EW), Accredited Social Health Worker (ASHA)/Auxiliary Nurse Midwife(ANM) Anganwadi worker(AWW)-ASHA/ANM &AWW are Government health workers situated in villages as per population guidelines and provide preventative maternal, child and other health services

Results

Results for Determinants of SHG Membership

Table 3 presents results from three separate stepwise forward logit regression models (Models I, II & III from second to the fourth column). The individual models fit were estimated using the maximum likelihood estimation method. The Odds Ratio (OR) values were reported, as measures of the strength of association, along with their associated 95% confidence intervals (95%CI) and significant p-value showing 0.01***,0.05** and 0.10* levels. Log-likelihood and

AIC/BIC values were also reported. Details of the three individual models are presented as follows.

Model I (Economic determinants of SHG Membership)

The results showed that odds of membership for poor households with a Below Poverty line Card (BPL) were 1.42 times higher than households without BPL cards or economically better households (95%CI: 1.22-1.64, p<0.01). The wealth quintile variable constructed for this paper using the surveyed households asset data shows that the odds of membership consistently reduced as the wealth quintiles descend from the marginally poor with the poorest (quintile five) 15 per cent less likely to be a member (OR = 0.85, 95%CI: 0.75-0.97, p<0.01) as compared to marginally poor (quintile one) households. However, the interaction of households(HH) with BPL card with wealth quintile, shows that the likelihood of membership for those households with a BPL card increases as the wealth quintiles decreases with the poorest Households(HH) with access to BPL cards almost 32 per cent more likely to be SHG members as compared to marginally poor households without BPL cards (BPL# Quintile five: OR = 1.32, 95%CI: 1.06-1.63, p<0.01). Additionally, eligible women presently working to earn in cash or kind are 32 per cent more likely to be members than non-working women (OR = 1.32, 95%CI: 1.20-1.45, p<.01).

Model II (Economic and Sociodemographic determinants of SHG Membership)

In model II, the inclusion of sociodemographic and health variables to the model I's economic determinants broadly maintained the direction and strength of associations previously observed in the first model. In Model II, membership likelihood increases to 73 per cent for BPL households as compared to 42 per cent in Model 1 (OR = 1.73, 95%CI: 1.41-2.13, p<0.01).

While the association of decreasing wealth quintile (marginally poor to poorest) and membership remained decreasing, the statistical significance for the poorest (fifth quintile) is now lost in model II. Moreover, the results also show the reduced magnitude and level of significance for the association of the poorest households (with BPL cards) and SHG membership

Model II results also showed that membership is higher for joint/extended families as compared to nuclear (OR = 1.32, 95%CI: 1.22-1.42, p<0.01), and also higher for Hindu compared to Muslim households (OR = 1.19, 95%CI:1.06-1.35, p<0.01). Eligible women belonging to Scheduled Caste, a proxy for social marginalization, are 38 per cent more likely to be members than women from the majority Hindu general caste (OR = 1.38, 95%CI: 1.22-1.53, p<0.010). Other marginalized social classes, the scheduled tribes and other backward classes were also more likely to be members, but their association is not significant. Individually, the impact of increasing level of education on the likelihood of membership shows positive association with women who completed primary education almost 23 per cent more likely to be a SHG member or belong to SHG households as compared to a woman with no schooling (OR = 1.23, 95%CI: 1.14-1.34, p<.01and women with secondary education (up to year 10) and above education 1.17 times more likely to be members, as compared to women with no schooling (OR = 1.17, 95%CI: 1.06-1.31, p<0.01). However, increasing the level of husband’s education is found to have a negative association with membership. Women whose husband completed secondary education and above are 11 per cent or 0.89 times *less likely* to be members as compared to women whose husband have no schooling (OR = 0.89, 95%CI: 0.80-1.00, p<0.05).

Model II also shows that membership associations with health and fertility indicators. The results show that in general, the likelihood of membership increases as the number of

pregnancies carried by women to gestation age(parity) increases (OR = 1.12, 95%CI: 1.08-1.17, p<0.01), and increases also when the total number of pregnancy loss rises ((OR = 1.09, 95%CI: 1.04-1.13, p<0.01).

Model III (Economic, Sociodemographic & area-level determinants of SHG Membership)

In model III, area and program-level variables are included. The associations observed previously in models (I & II) for economic and sociodemographic variables with SHG membership remain the same but with two notable exceptions. The first is that the association of membership for women from households in the fifth wealth quintile or the poorest in this sample becomes significant again and shows that they are almost 30 per cent or 0.70 times *less likely* to be members as compared to marginally poor households in the same villages (OR = 0.71, 95%CI: 0.60-0.85, p<0.01). On the contrary, the use of BPL indicator shows that Households with BPL cards are still 70 per cent or 1.70 times more likely to be SHG members as compared to HH with no BPL cards(OR=1.70,95%CI:1.37-2.11,p<0.01)

Examining the effects of area-level variables included in this model, the results show that the likelihood of SHG membership increases for women in villages with a higher number of community health workers (OR = 1.06, 95%CI: 1.04-1.08, p<0.01). Similarly, SHG membership increases for women in villages with the increasing number of contacts with a community health worker(ASHA/AWW/ANM) or SHG member in the eligible woman's last pregnancy (OR = 1.01, 95%CI: 1.01-1.02, p<0.01). Moreover, the model shows the effects of loan purpose, a program variable, that is related to the household's last reason for borrowing money, and the effect of interacting loan purpose with wealth quintile. The model shows that the likelihood of membership is higher when households borrow loans for enterprise purposes, for example, to start or expand business (OR = 8.59, 95%CI: 5.15-14.31, p<0.01) as compared

to households that did not borrow any loans in the last 3 years. Similarly, membership is also higher when borrowing for non- enterprise reasons such as education or marriage (OR = 2.26, 95%CI: 1.61-3.17, p<0.01) as compared to non-borrowing households. Interestingly, the likelihood of membership *decreases* when the purpose of loans borrowed is for a *health purpose,* for example, to meet the cost for illness, delivery expense (OR = 0.74, 95%CI: 0.59-0.91, p<0.01 compared to non-borrowing households. Sources of borrowing for Non-SHG members include family/friends/banks/ informal money lenders, but not through SHGs

The link between access to finances, poverty and health is also observed in model III from the interaction of quintile with loan purpose. This interaction shows that the poorest (quintile five)households who borrowed loans for health expenses are 1.85 times more likely to be members as compared to the poor(quintile one) who didn't take any loans (OR = 1.85, 95%CI: 1.37-2.48, p<0.01)

One plausible hypothesis presented here suggests that loans borrowed for health reasons divert funds from potential enterprise activities, the analysis shows that by interacting round with loan purpose, those households in round 2 who borrowed loans for health reasons were almost 0.64 times *less likely* to be SHG members than those who didn't borrow any loans in round 1 (OR = 0.64, 95%CI: 0.49-0.72, p<0.01).

Results for Determinants of Duration of membership in SHGs

The results from the hurdle negative binomial (HNB) regression model are presented in Table 3 (last column) with the Incidence Rate Ratio (IRR) values reported along with their associated 95% confidence intervals (95%CI). The explanatory variables used in this model are the same as for the three logit regression models for the SHG membership analysis.

HNB Regression results for Duration of Membership in SHG

The results show that once enrolled into SHGs, women belonging to households with below poverty line (BPL) cards are 1.12 times more likely to remain, members, as compared to households without BPL cards (IRR = 1.12, 95% CI: 1.01-1.23, p <0.05). The likelihood of staying members (duration) increases across the poverty gradient represented by the wealth quintile with the poorest (quintile5) households being 1.44 times more likely to stay members as compared to the poor (IRR = 1.44, 95%CI:1.30-1.58, p<0.01). The model also reveals that joint families as compared to nuclear families are more likely to stay SHG members for longer (IRR = 1.16, 95%CI: 1.11-1.20, p<0.01). Conversely, poor households belonging to disadvantaged social class (scheduled castes), compared to general caste, are less likely to stay members (IRR = 0.87, 95%CI: 0.82-0.92, p <0.01).

The levels of education attained by women and their husbands have opposite effects on duration. While women with secondary education and above are **less** likely to continue membership (IRR = 0.89, 95%CI: 0.84-0.93, p<0.01) as compared to women with no schooling. Eligible women whose husbands completed secondary education or above are more likely to continue membership for longer compared to women whose husband's had no schooling (IRR = 1.11, 95%CI:1.04-1.1, p<0.01).

Results from other sociodemographic and health variables in model III show that while women who reported a higher number of pregnancy loss are more likely to stay members (IRR = 1.02, 95%CI:1.00-1.03, p<0.05), women with increasing number of pregnancies(parity) were *less* likely to stay members, however, the association for parity is not significant (p >0.10).

Model III also shows the effects of area-level factors on SHG duration. The findings show that women in villages where both government and private health facilities were available are more likely to continue membership for a longer period(IRR=1.06, 95%CI: 1.00-1.12,p<0.05)

As the second round of survey data collection was completed after two years of program implementation in 2017, the model finds that households interviewed in round 2 are 2.73 times more likely to be members compared to those interviewed in round1 (2015) when program implementation (health layering) was yet to begin (IRR = 2.73, 95%CI: 2.61-2.86, p<0.01).

The results also show that households that borrow loans for *any* purpose are more likely to stay members for longer, with those borrowing for health reasons being 1.50 times more likely to stay members (IRR = 1.47, 95%CI: 1.36-1.62, p<0.01) as compared to those households who do not borrow any loans. Model III also includes the interaction of wealth quintile with loan purpose(quintile # loan purpose) to examine the effect on duration in SHGs among gradients of poor and the different reasons they borrow money the last time from any source (bank, moneylender, family/friends/neighbour). The interaction results show that poorer households, in fourth and fifth quintile, are more likely to remain SHG members when borrowing loans for enterprise purposes as compared to poor households (quintile1) that did not borrow any loans (IRR = 1.17, 95%CI: 1.01-1.35, p<0.05).

Table 3.Showing Odds Ratio estimates for various Logit Analysis for Determinants of SHG Membership(Model I-III) and Incidence Rate Ratio estimates for Hurdle Negative Binomial Regression Analysis for Determinants of SHG Duration Model(III)*

		**SHG Membership Logit Analysis**			**SHG Duration: Hurdle Negative Binomial Analysis**
**S.no**	**Variable name**	**Model I (Economic variables only)**	**Model II (Economic + Sociodemographic variables)**	**Model III (Economic+ Sociodemographic+ Area Variables)**	**Model III (Economic + Sociodemographic + Area Variables)**
		OR (95% CI)	OR (95% CI)	OR (95% CI)	IRR (95% CI)
1	**Household (HH) has Below Poverty (BLP) Card**				
	No(reference)				
	Yes	1.42 *** (1.22-1.64)	1.73 *** (1.41 -2.13)	1.70*** (1.37-2.11)	1.12** (1.01-1.23)
2	**Quintile (rich to poor)**				
	1. Marginally Poor (reference)				
	2. Moderately Poor	1.03 (0.90-1.17)	1.04 (0.91-1.19)	0.93 (0.80-1.08)	1.10** (1.01-1.20)
	3. Poor	1.16** (1.01-1.32)	1.18** (1.02-1.35)	1.02 (0.87-1.20)	1.17*** (1.07-1.28)
	4. Poorer	0.97 (0.85-1.11)	1.02 (0.88-1.19)	0.83** (0.70-0.98)	1.26*** (1.15-1.37)
	5. Poorest	0.85** (0.75-0.97)	0.94 (0.80-1.10)	0.71*** (0.60-0.85)	1.44*** (1.30-1.58)

		SHG Membership Logit Analysis			SHG Duration: Hurdle Negative Binomial Analysis
**S.no**	**Variable name**	**Model I (Economic variables only)**	**Model II (Economic + Sociodemographic variables)**	**Model III (Economic+ Sociodemographic+ Area Variables)**	**Model III (Economic + Sociodemographic + Area Variables)**
		OR (95% CI)	OR (95% CI)	OR (95% CI)	IRR(95% CI)
3.	**HH has Below Poverty Card # Quintile (Rich to Poor)**				
	BPL card (Yes)# Quintile 1 (Marginally Poor)(reference)				
	BPL(Yes) #Quintile 2 (Moderately Poor)	0.96 (0.80-1.21)	0.93 (0.76-1.14)	0.92 (0.75-1.15)	0.89*** (0.80-0.97)
	BPL(Yes) # Quintile 3 (Poor)	0.98 (0.79 -1.20)	0.92 (0.75-1.14)	0.94 (0.76-1.16)	0.97 (0.87-1.07)
	BPL(Yes) # Quintile 4 (Poorer)	1.18 (0.94-1.43)	1.10 (0.89-1.37)	1.07 (0.86-1.33)	0.88** (0.80-0.98)
	BPL(Yes)# Quintile 5 (Poorest)	1.32*** (1.06-1.63)	1.23* (0.99-1.53)	1.11 (0.89-1.40)	0.89** (0.80-0.99)
4	**Eligible Woman Presently Working status**				
	Not Working (reference)				
	Presently Working to earn in cash, kind or both	1.32*** (1.20-1.45)	1.25*** (1.14-1.38)	1.26*** (1.15-1.40)	0.98 (0.94-1.03)

		SHG Membership Logit Analysis			SHG Duration: Hurdle Negative Binomial Analysis
**S.no**	**Variable name**	**Model I (Economic variables only)**	**Model II (Economic + Sociodemographic variables)**	**Model III (Economic+ Sociodemographic+ Area Variables)**	**Model III (Economic + Sociodemographic + Area Variables)**
		OR (95% CI)	OR (95% CI)	OR (95% CI)	IRR(95% CI)
5	**Type of Family**				
	Nuclear Family (reference)				
	Joint & Extended Family		1.32*** (1.22-1.42)	1.30*** (1.20-1.40)	1.16*** (1.11-1.20)
6	**Religion (Household Head)**				
	Muslim Household(ref)				
	Hinduism & Others		1.19*** (1.06-1.35)	1.21*** (1.07-1.38)	0.97 (0.91-1.03)
7	**Caste**				
	General Hindu Caste(ref.)				
	Other Backward Caste		1.04 (0.94-1.16)	0.97 (0.88-1.09)	0.93*** (0.88-0.99)
	Scheduled Tribe		1.02 (0.87-1.21)	1.07 (0.90-1.28)	0.90** (0.82-0.99)
	Scheduled Caste		1.38*** (1.22-1.53)	1.30*** (1.16-1.46)	0.87*** (0.82-0.92)

		SHG Membership Logit Analysis			SHG Duration: urdle Negative Binomial Analysis
**S.no**	**Variable name**	**Model I (Economic variables only)**	**Model II (Economic + Sociodemographic variables)**	**Model III (Economic+ Sociodemographic+ Area Variables)**	**Model III (Economic + Sociodemographic + Area Variables)**
		OR (95% CI)	OR (95% CI)	OR (95% CI)	IRR(95% CI)
8	**Eligible woman (EW) Age in years**		1.01*** (1.01-1.02)	1.01** (1.00-1.02)	1.01*** (1.01-1.02)
9	**Eligible woman education level(completed years)**				
	No schooling(reference)				
	Completed Primary& Middle School(up to year 9)		1.23*** (1.14-1.34)	1.17*** (1.07-1.27)	0.96 (0.93-1.01)
	Completed Secondary(up to year 10) and Above		1.17*** (1.06-1.31)	1.08 (0.97-1.20)	0.89*** (0.84-0.94)
10	**EW's Husband Education level(completed)**				
	No schooling(reference)				
	Completed Primary& Middle School(up to year 9)		1.10** (1.00-1.21)	1.08 (0.98-1.19)	1.06*** (1.01-1.11)
	Completed Secondary(up to year 10) and Above		0.89** (0.80-1.00)	0.90* (0.80-1.01)	1.11*** (1.04-1.17)

		SHG Membership Logit Analysis			SHG Duration: Hurdle Negative Binomial Analysis
**S.no**	**Variable name**	**Model I (Economic variables only)**	**Model II (Economic + Sociodemographic variables)**	**Model III (Economic+ Sociodemographic+ Area Variables)**	**Model III (Economic + Sociodemographic + Area Variables)**
		OR (95% CI)	OR (95% CI)	OR (95% CI)	IRR(95% CI)
11	**Parity**		1.12*** (1.08-1.17)	1.10*** (1.06-1.15)	0.98 (0.97-1.00)
12	**HH Below poverty Card # Parity**				
	No(ref)				
	Yes card# Parity		.90*** (0.86-0.95)	0.89*** (0.85-0.94)	1.00 (0.97-1.00)
13	**Total Pregnancy Loss**		1.09*** (1.04-1.13)	1.05** (1.00-1.09)	1.02** (1.00-1.03)
15	**Place of Last Delivery**				
	Home Delivery(reference)				
	Institutional Delivery			1.10 (0.98-1.23)	1.07** (1.00-1.16)

		SHG Membership Logit Analysis			SHG Duration: Hurdle Negative Binomial Analysis
**S.no**	**Variable name**	**Model I (Economic variables only)**	**Model II (Economic + Sociodemographic variables)**	**Model III (Economic+ Sociodemographic+ Area Variables)**	**Model III (Economic + Sociodemographic + Area Variables)**
		OR (95% CI)	OR (95% CI)	OR (95% CI)	IRR(95% CI)
15	**Availability of Health facility in the village**				
	No Health Facility(reference)				
	Only Government Health Facility			0.95 (0.88-1.03)	1.03 (0.99-1.07)
	Only Private Health Facility			0.96 (0.72-1.27)	1.03 (0.91-1.15)
	Both Government & Private Health Facility			0.96 (0.83 -1.10)	1.06** (1.00-1.12)
16	**Total Number of Private Doctor Clinics in village**			1.01 (0.97-1.07)	0.99 (0.98-1.01)
17	**Number of community health workers(ASHA & ANM) in the village**			1.06*** (1.04-1.08)	0.98*** (0.98-0.99)
**18.**	**Number of contact with ASHA/ANM/AWW/ SHG in last pregnancy**			1.01*** (1.01-1.02)	1.00 (1.00-1.00)

		SHG Membership Logit Analysis			SHG Duration: Hurdle Negative Binomial Analysis
**S.no**	**Variable name**	**Model I (Economic variables only)**	**Model II (Economic + Sociodemographic variables)**	**Model III (Economic+ Sociodemographic+ Area Variables)**	**Model III (Economic + Sociodemographic + Area Variables)**
		OR (95% CI)	OR (95% CI)	OR (95% CI)	IRR(95% CI)
19.	**Last Loan Purpose**				
	No Loan availed(reference)				
	Enterprise reasons			8.59*** (5.15-14.31)	1.55*** (1.37-1.74)
	Non- Enterprise reasons			2.26*** (1.61-3.17)	1.91*** (1.68-2.16)
	Health & Illness			0.74*** (0.59-0.94)	1.47*** (1.36-1.62)
	Others(Reason not stated)			1.00 (0.67-1.49)	1.60*** (1.37-1.87)
20	**Evaluation Round**				
	Round1(reference)				
	Round 2			1.57*** (1.43-1.71)	2.73*** (2.61-2.86)

		SHG Membership Logit Analysis			SHG Duration: Hurdle Negative Binomial Analysis
**S.no**	**Variable name**	**Model I (Economic variables only)**	**Model II (Economic + Sociodemographic variables)**	**Model III (Economic+ Sociodemographic+ Area Variables)**	**Model III (Economic + Sociodemographic + Area Variables)**
		OR (95% CI)	OR (95% CI)	OR (95% CI)	IRR(95% CI)
21	**Round# Last Loan Purpose**				
	Round 1#NoLoan (reference)				
	Round 2# Loan for Enterprise reasons			0.41*** (0.29-0.56)	0.56*** (0.51-0.62)
	Round 2#Loan for Non – Enterprise reasons			0.72*** (0.57-0.93)	0.59*** (0.53-0.65)
	Round 2#Loan for Health& Illness reasons			0.64*** (0.49-0.72)	0.63*** (0.57-0.70)
	Round 2# Loans for Other purposes			3.64*** (2.11-6.26)	0.61*** (0.53-0.69)
22	**Quintile (Rich to Poor) #Last Loan Purpose**				
	Quintile 1 #No loans (ref.)				
	Quintile 2 #Enterprise Reasons			0.73 (0.41-1.28)	1.01 (0.87-1.17)
	Quintile2#NonEnterprise Reasons			0.99 (0.65-1.48)	0.94 (0.81-1.10)

S.no	Variable name	SHG Membership Logit Analysis			SHG Duration: Hurdle Negative Binomial Analysis
		**Model I (Economic variables only)**	**Model II (Economic + Sociodemographic variables)**	**Model III (Economic+ Sociodemographic+ Area Variables)**	**Model III (Economic + Sociodemographic + Area Variables)**
		OR (95% CI)	OR (95% CI)	OR (95% CI)	IRR(95% CI)
	Quintile 2#Health Reasons			1.22 (0.92-1.62)	1.14** (1.01-1.29)
	Quintile 2#Other Reasons			2.3*** (1.47-3.71)	0.96 (0.82-1.12)
	Quintile 3 #Enterprise Reasons			0.79 (0.45-1.42)	1.07 (0.92-1.24)
	Quintile3#NonEnterprise Reasons			0.81 (0.55-1.21)	0.93 (0.80-1.08)
	Quintile 3#Health Reasons			1.40*** (1.05-1.87)	0.99 (0.93-1.21)
	Quintile 3# Other Reasons			2.51*** (1.57-4.00)	0.94 (0.80-1.11)
	Quintile 4 #Enterprise Reasons			0.77 (0.43-1.35)	1.17** (1.01-1.35)
	Quintile 4#NonEnterprise Reasons			1.08 (0.72-1.61)	1.01 (0.88-1.17)
	Quintile 4#Health Reasons			1.85*** (1.37-2.48)	1.06 (0.94-1.21)

		SHG Membership Logit Analysis			SHG Duration<br>Hurdle Negative Binomial Analysis
**S.no**	**Variable name**	**Model I (Economic variables only)**	**Model II (Economic + Sociodemographic variables)**	**Model III (Economic+ Sociodemographic+ Area Variables)**	**Model III (Economic + Sociodemographic + Area Variables)**
	Quintile 4#Other Reasons			1.85***<br>(1.36-2.50)	0.93<br>(0.78-1.10)
	Quintile 5 #Enterprise Reasons			0.71*<br>(0.41-1.23)	1.16**<br>(1.00-1.35)
	Quintile 5#NonEnterprise Reasons			1.60**<br>(1.06-2.42)	0.89<br>(0.77-1.04)
	Quintile 5#Health Reasons			2.04***<br>(1.46-2.85)	0.95<br>(0.81-1.12)
	Quintile 5#Other Reasons			3.64***<br>(2.11-6.26)	0.82**<br>(0.68-0.99)
	**Estimation of Model Fit**	**SHG Membership(Logit)**			**SHG Duration(HNB)**
		**Model I**	**Model II**	**Model III**	**Model III**
	Log likelihood	-10,464	-10,333	-9,952	-45,704
	Number of Observation	15,300	15,300	15,300	15,300
	AIC/BIC	20,950 21034	20,715 20,898	20,016 20,444	91,635 92,497

Note Confidence intervals in parentheses; ***p<0.01; **p<0.05; *p<0.10; Determinants of SHG Membership using Three step by step forward Multivariable Logit Regression Models; Determinants of SHG Duration using a Hurdle Negative Binomial (HNB) Regression Models; Assessment of Economic, Socio-Demographic, and area-level explanatory variables in both analyses

May be really useful for authors to pick model 3 to report in paper and refer to other two models if required in discussion, rather than present all three models in results and in discussion This may significantly improve clarity of results and discussion section. Also may be useful to carefully consider the value of all the variables in model 3.

Discussion

This study makes a significant contribution to the literature in investigating the determinants of membership and duration of membership of integrated health literacy and microfinance based SHG program in India. One recent study (15) from India looked at how microfinance only SHGs evolved over time using a survival analysis model. That study used information on women who had been members of SHG at any stage, not necessarily the continuing members. The study was based on five locations in North India, excluding Uttar Pradesh, the largest and the most populated state. The study found higher rates of exiting/departure from SHG up to 20 per cent among disadvantaged castes and households without land holdings and found a protective effect of some formal education on duration. Our analysis, used more a sophisticated, a modified count data model to examine the factors that determine how long a woman/household remained an SHG member among 15,300 women among which 53 per cent were current SHG members in Uttar Pradesh. Moreover, by analysing determinants of membership and duration of membership in one integrated model, this study provides evidence to guide SHGs programs to improve their program designs to retain clients. Furthermore, focussing on duration in addition to membership is an important consideration as recent research on microfinance effectiveness recommend programs supporting poor households for longer duration till they are able to move up to the next rung of poverty (22). The focus on the duration of membership, therefore, echoes a commitment to assist SHGs in identifying the right 'poor' clientele for membership and then help retain them as members till they cross the poverty threshold at a minimum. Moreover, this study also improves on previous study designs by using multiple (22) number of explanatory variables to explains much of the variation with the outcome variables

More importantly, the study shows that poor households with Below Poverty Line cards are more likely to be SHG members as compared to poor households without BPL cards. Contrastingly, the study also finds that the poorest households based on the wealth quintile constructed as part of this study using household assets (quintile 4 and 5) are less likely to be SHG members. The findings from the wealth quintile support previous evidence from regional India that showed exclusion of the poorest of the poor in typical microfinance programs (15,16,18-20,34,35). Programs such as the specialized SHG plus health literacy model

evaluated in this research that primarily seeks to target the poor and poorest are adequately able to capture the poor as demonstrated through the BPL statistics. However, in order to ensure that the poorest are also captured in the program requires the use of additional measures such as through wealth quintile demonstrated here.

These issues essentially highlight potential targeting gaps that other programs using similar(SHG) model may be exposed to when the poorest are identified through wealth-based quintiles in contrast to income-based economic variables such as the BPL cards alone. The research also adds new evidence on what factors influence continued membership or ‘duration’. Our analysis shows that the likelihood of continuing as members in SHG (duration) increases for poor households whether they are captured through BPL or the wealth quintile. The poorest captured through the wealth quintile are 1.43 times more likely to continue as members as compared to relatively better-off or marginally poor households, and poor households with BPL cards 1.12 times more likely to continue as members as compared to those without BPL cards. This suggests all gradients of the poor, especially the poorest potentially gain from membership when provided membership opportunity.

This study shows that poor households with Below Poverty Line(BPL) cards are 70 per cent more likely to join and 12 per cent more likely to stay members demonstrating the programs ability to capture these poor households. BPL is an income limit threshold used by the Indian government to identify poor households eligible for entitlements when the annual income is less than USD 376 (Rs 26,000) per annum (36). In spite of BPL being the preferred economic measure in use by the Indian government, the use of BPL cards as an effective criterion to capture the poor is widely debated.

Challenges associated with BPL have been highlighted (27,28) and an Indian government report (37) found that up to half of all eligible poor households from rural India were missed due to BPL targeting issues. Limitations are also recognized by Indian federal poverty alleviation program, the National Rural Livelihood Mission that uses a Participatory Inclusion Process to identify clientele instead of using BPL cards (38). Some studies found that richer households exploited targeting errors in the BPL inclusion criteria to get a BPL card and be eligible to receive associated entitlements. Similar inferences regarding use of BPL cards and households with BPL cards can be applied to the context of Uttar Pradesh state, and the program population

analysed in this paper. Therefore, this paper adds significant value to the literature on potential targeting gap for the poverty alleviation programs in India by constructing and using an additional wealth quintile to capture poverty and its gradients beyond BPL status. Both BPL and wealth quintile are used as explanatory variables in this paper to allow their comparison in whether the poor are captured in the program.

Most smaller studies (15,16,18-20,35,36) from regions of India have previously highlighted the microfinance's limitation in effectively reaching the poorest These studies have mainly relied on households' land holding, sources of income and amount earned to identify the poorest bypassing the use of formal measures of BPL and constructed wealth quintiles as done in this paper.

Some discussion on practicality of using wealth quintiles for government programs would be useful. It is an easy method to use rather generally used for analysing a program in a study, but tough to be used as a method for identifying poor in national programs.

Targeting the Poorest from the Lowest Wealth Quintile Households in the SHG plus Health Literacy Program in Uttar Pradesh

Previous studies showed that traditional microfinance models mostly served poor clients that were at or just below the poverty line (2,10,15). A specialized type of microfinance, SHGs, in contrast, are considered better suited in reaching the poorest *within* the BPL population, as SHGs inherently operate on a pro-poor developmental agenda (10). However, studies elsewhere showed that the poorest can still be excluded from some SHGs due to reasons related to caste-based social marginalization, lack of minimum household finances to join groups, and intrinsic biases of implementing organisations preferring to enrol better off populations that are more easily reached (3, 11,16). SHGs by design focus on inclusion of poor clients at the *start* of programs with the assumption that once enrolled, the selected clients stay members. Similar to other studies (15, 16), this paper finds that the poorest when identified through the wealth quintile (but not through the BPL variable) are less likely to be SHG members; however, the analysis generates new evidence for the state of UP showing that once poorer households do join SHGs, they are likely to stay members for longer. While the poorest represented by the last or fifth wealth quintile in this paper are almost 30 per cent *less likely* to be SHG members at the start, when they do join, they are 40 per cent *more likely* to continue as members as compared to better-off households. This suggests that the benefit of joining SHGs may become evident for poorer households only after they join SHGs and that programs can use this while communicating the benefits of membership to potential clients comprising poorer household.

very important policy implcations

This conclusion is also shown in this paper through the examination of membership and duration of membership using the Below Poverty Line (BPL) variable, that shows poor households with a BPL card are more likely to be SHG members as well as continue longer as members as compared to poor households without BPL cards.

In order to analyse the effect of BPL card status among various categories of the poor, we have also interacted BPL with wealth quintile. It is possible that the wealth quintile constructed for this research is better able to capture/represent the poor in rural India compared to the BPL card status since the former involves multiple household assets and facilities. It is however interesting to note that the results from the interaction of BPL # quintile show the likelihood of continuing as SHG member(duration) decreases as we move down the wealth quintiles of poor households that have a BPL card as compared to households without BPL cards. This may be due to BPL cards failing to reach the poorer households in these villages, that are better captured by our wealth quintile. The use of broader criteria such as the wealth quintile constructed in this study is supported in the literature that recommends identifying the poorest using multidimensional indicators of deprivation that are better able to capture the varied manifestations of poverty (27, 28).

Effect of Borrowing on SHG Membership and Duration.

Microfinance is based on the premise that poor households can improve their social outcomes when they are provided accessible and affordable financial services for investing in income generation activities but also for managing economic shocks and repaying loans at low-interest rates (3, 4,9,11) However, pragmatic views on microfinance highlighted that not all poor borrowers could generate profit from loans as not everyone has the necessary entrepreneurial skillset to turn around adversity into profit. Moreover, limited enterprise opportunities in rural markets influence economic opportunity in the first place for poor households (2,6). Accurate estimation of how much and whether households gain from membership is limited without additional time-series data on household's movement out of poverty. The current study instead used an explanatory proxy variable(loan purpose), to study membership and duration effects looking at the reasons for which households borrowed their last loan when more than USD7(Rs 500) was borrowed. Four categories (Enterprise/Non-enterprise/Health/Others-reason not stated) are created to classify the reasons for borrowing the last loan both by SHG or Non-SHG

HH from any source. The results show that when women borrowed funds for any purpose (except for health), they are more likely to become SHG members and continue membership for longer period. However, the results show that when women or households borrowed funds for health reasons such as delivery expense or treatment of illness, they were 26 percent less likely to become SHG members. Yet, the results also show that SHG members are almost 47 per cent more likely to continue as members when they borrow for health reasons. While SHGs provide loans that can primarily be used for poverty reduction through investing in income-generating activities, loans borrowed for health expenses potentially divert funds from enterprise opportunity. The burden of borrowing for health reasons in this sample is also shown in table 2 by being the most common reason why SHGs(15 percent) and non SHG(12 percent) borrowed the last loan The variable representing borrowing for health reason, exerts opposing effects on membership (negative influence) and duration (positive influence). This may be due to limited awareness among non-members who may only consider SHG membership for enterprise reasons.

This may also suggest that households can *initially* be reluctant to view SHGs as a potential source of funds to meet health expenses, potentially discouraging them from joining SHGs. However, we also find that borrowing for any reasons (including health), as seen in the interaction of survey round with loan purpose, is negatively linked to the duration of membership in SHG. The exact mechanism explaining why the duration is negatively affected is not well understood, but as mentioned previously, the literature suggests that over-indebtedness is a recognized issue in microfinance with underlying any reason especially for health care expenses, a stimulus for repeat borrowing (9,11,41,42).

The effect of loan purpose among different gradients of the poor on membership and duration are also evaluated in this paper by interacting last loan purpose with wealth quintile (Loan purpose # quintile). The results show that poorer households in quintiles 3, 4 and 5, in contrast to poor households, are more likely to *become SHG members* when they borrowed for health-related expenses with poorest households in quintile 5 more likely to be members when also borrowing for non-enterprise reasons. However, once these households become members, the likelihood of *staying members* is observed only when poor(er) households (quintiles 4,5) borrowed for enterprise or income-generating activities and not for health expenses.

These preferences and associations indicate that in the rural Uttar Pradesh context, poorer households may be turning to SHGs to borrow money for health reasons which help them tide over health-related expenditures. Studies from India find that vulnerable households at the threshold of poverty get pulled deeper into poverty when exposed to maternal and newborn health issues due to lack of affordable financial sources to pay for treatment that is mainly Out of Pocket (41,42). Therefore, households that receive financial reprieve through SHGs may be in a better economic situation to initially build household savings and reserves that permit them to participate in income-generating or enterprise activities as continuing members of SHGs.

Effect of education on SHG membership and duration

Beyond economic indicators, our research also shows that increasing levels of education of eligible women and their husbands had contradicting effects on membership and duration. For women, being educated is seen to be associated with a higher likelihood of membership but not with duration, suggesting that more educated women may have a *greater* individual agency to try an initiative but choose not to continue if they do not derive benefit. Contrary to the effects of women's education, the results show women whose husbands are more educated were initially *less likely to be members*, but once they joined, they were significantly more likely to remain members. A likely explanation is that educated husbands are not initially supportive of their wives in joining SHGs due to limited awareness or understanding of how membership may translate into household improvement. We see that levels of education have a differing effect on the duration of membership suggesting differences in how more educated women do (or do not) utilise microfinance. Although this reasoning does not adequately explain why more educated husbands would prefer that their wives stay members as found in our study. One line of reasoning worth pursuing relates to more educated men leverage their wives to borrow funds for their businesses, or for purposes where the wife has little say on loan usage (14,40,43). However, since the woman herself is the primary borrower in the SHG and the responsibility of loan repayment rests with the woman, an educated, empowered woman may be less willing to borrow funds in her name when her husband controls the use of borrowed funds as also observed in previous studies(14,40,43).

Higher education for women in our study does not, however, play a protective role in duration of membership, contrary to another study (15) from India, which found that women with some

education were 30 per cent more likely to remain, members and that SHGs, where all members were educated, were more likely to survive longer. In our study, women's secondary or higher education status was negatively associated with duration of the membership. This may be due to more educated women with higher education (a proxy for better-off households) preferring non-microfinance-based sources for credit. Our result in this regard is supported from another study in India that found better-off households (representing higher educated members) preferred borrowing from informal credit sources over microfinance and were more comfortable in directly navigating the formalities associated with banks (14,16).

Impact of Social class (caste) on SHG Membership and Duration

Previous studies from different states of India found that women belonging to scheduled caste were more likely to be members in SHG only programs (14, 16,25). Our results support this evidence and show that membership for women belonging to scheduled caste (SC) is higher even in the Integrated SHG and health literacy program evaluated in this research. We, however, find that women belonging to scheduled caste and tribes are *less likely to continue as* SHG members as compared to general Hindu caste members. While previous studies have found a similar association (14,16,25), our study extends this association of reduced likelihood of continuing members(duration) in rural Uttar Pradesh but also in the context of integrated microfinance and health literacy program. We also test for the independent effect of caste alone on duration (results not reported here) using an appropriate count data model. We find an independent and significant negative effect of caste on the *duration of membership in SHG*. Studies elsewhere have also shown in some instances that upper caste members are more likely to *join* SHGs and remain members of the groups they joined (15). Our study found the same effect for women in rural Uttar Pradesh with SC/ST and OBC households almost 13 per cent *less likely to continue as* members as compared to non-scheduled or general caste Hindu women. Among SC/ST castes, our study results are similar to previous evidence that found households belonging to Scheduled Tribes (ST) more likely to leave SHGs as compared to SC (15). This is pertinent in Uttar Pradesh which predominant has a larger SC than ST population, suggesting that community programs in UP may need strategies to support both SC and ST households in staying members (23).

Religion and SHG membership

We also evaluated another key social factor, religion, that governs many social norms in rural India. This study found that women from households where the head of the family identified themselves as being Hindu, were 1.19 times more likely to become SHG members as compared to Muslim households(HH). While Hindu HH compared to Muslims HH were less likely to *continue as* SHG members, the association is not statistically significant. Religion as a determinant in membership and duration is important considering that community groups such as SHGs present opportunity to reach HH belonging to different minority religions(Islam, Sikhism, Christianity etc) in India. The largest minority religion in India, Islam comprise 13 per cent of the national population and 19 per cent of UP's population and in some instances has encountered higher social exclusion and deprivations (44–46). Thus, India's current federal poverty alleviation program, the NRLM that uses a similar SHG model as evaluated in this paper presents an opportunity to focus social policy to extend benefits of memberships in SHG program to all vulnerable communities groups.

Maternal health Indicators and SHG membership and Duration

The SHG program implemented in rural UP and analysed in this paper also had an added maternal health literacy component. The current study finds that an increasing number of previous pregnancies(parity) is associated with a higher likelihood of membership. While SHGs usually comprise of older women that may have completed their families, the inclusion of parity permits analysis of membership behaviours separate from the eligible woman's age. This finding contrasts with another study from a different part of India which found that parity was negatively associated with membership especially if the mother was already a carer of an under-five-year-old (14). However, there is a fundamental program design difference between the previous study and the current study. While the previous study looked at a microfinance only *intervention*, our study i.e. the UP program investigates the effect of a microfinance intervention with an added maternal health literacy component. As observed in the UP program, the higher membership association of parity may be due to the additive effect of the added health literacy program. The health literacy sessions added to regular SHG meetings offer women with maternal health knowledge and may be acting as an incentive for women in favour of SHG membership. SHGs by design provide a platform for discussion and knowledge sharing,

Please refer to my question re mean age.

and mothers may be more likely to join groups with the added value of a health literacy component. The peer effect in SHG groups other than access to credit, for example, was recently attributed by a study in India that showed increased school enrolment of members children as compared to non-members (47)This is pertinent in the rural UP context where almost 70 per cent of women lack access to traditional sources of information through the media (TV, Radio, newspaper) coupled with gaps in the quality of health information provided through the public health system (48,49). The membership appeal in SHGs for women and mothers can thus be understood. Moreover, a previous study from India showed an inverse relation of increasing parity and health care utilization, potentially re-exposing women with a higher number of pregnancies to maternal health risks(50). Therefore, membership in SHGs and the associated peer effect may positively influence desired health behaviours, especially among women with higher parity. Therefore, adding a health intervention to a microfinance program seems to strengthen the model overall by encouraging membership, and may offer evidence in favour of similar integrated programs being scaled up. Our findings support adding health intervention to India's federal poverty alleviation plan the N.R.LM which currently reaches 620 million households and present an opportunity to accelerate towards the 2030 Sustainable Development Goals for health and poverty reduction (51).

Unclear if this study provides any such evidence re benefits of adding health intervention to SHGs. Suggesting that such integrated programs should be scaled up requires much clear evidence and information on cost-effectiveness, so may be best to avoid this here.

Health Service Availability and Microfinance

In the Indian health system, public health facilities are distributed according to population levels with the availability of primary health facilities indicative of rural residence or remoteness (52). Villages with any available health facility are considered better connected and therefore viewed as being less rural. In the program villages evaluated in our study, we find that women in villages with both government and private health facility had a longer duration of the membership. This may suggest that villages with health facilities may be less rural or better connected to roads and therefore more able to provide greater opportunity to engage in rural micro-enterprise for SHG members. This study also shows that a higher number of community health workers in a village, and more contacts with them during pregnancy *increases membership likelihood*, as does having an institutional delivery. However, only the association for the number of community health worker with SHG membership is statistically significant. Other studies (53) from the same project, previously reported that program implementation increased the social network and coordination between community health workers and the SHG

platform leading to an increased density of social network and transmission of health information. This suggests a pathway linking SHG membership and number of community health workers

Our study differed from past studies in the methodological rigour employed to assess determinants of membership and duration simultaneously. Indeed, the hurdle negative binomial model employed in this study is better suited for a SHG based model where membership and duration are both considered in the same model but analysed separately. Moreover, no large-scale study in the context of rural India primarily rural Uttar Pradesh has previously provided empirical evidence on the association of the poorest households and SHG membership/duration, importantly in relation to the availability of BPL cards amongst various gradients of the poor. Additionally, this study also fills a gap in the microfinance literature by triangulating the influence of poverty on membership and duration by using the availability of BPL cards, wealth quintile and different levels of the poor with BPL cards (by interacting BPL# wealth quintile)

Limitations

The key limitations of the study are highlighted. Although the same villages were visited in both survey rounds, different households were sampled in both rounds. The lack of temporal data prevents examination of determinants of continued membership following the same woman/household in both survey rounds. Additionally, it is possible that a few households may have been captured in both survey rounds, however, these would be a limited sample.

Another limitation is the possibility of recall bias, especially for women who were asked to remember details of the last loan taken within the last three years. The value of loan amount which differentiates non-borrowing and borrowing households i.e. 7 USD (Rs 500) may miss including households that routinely borrow smaller amounts.

Another limitation is in examining area-level effects across villages while there may potentially be clustering within villages. While the survey is cluster designed to sample households within villages or GPs, the sampling also, allowed selecting matched blocks, and villages within blocks that are expected to be broadly similar across crucial developmental and

social parameters. Consequently, the standard cluster sampling effects does not apply here and there seem to be more similarities across villages than within villages. However, in India health services and facilities are implemented at the level of the village and they are likely to vary across villages. Therefore, it makes more sense to study the effect of area-level health system variables such as health facility availability, number of community health workers across villages on SHG membership and duration.

Further, the way the outcome variable of SHG membership is designed, sampled women comprising SHG and Non-SHG, are selected from the same villages. However, in some cases, the women sampled in a village were either all members or all non-members because of which the outcome variables may not exhibit enough variation within a village. Therefore, it makes more sense to assess the effect on outcome variables across villages instead of within villages as the clustering within villages may be less likely to have an influence here.

Lastly, the study's cross-sectional design prevents concluding causality from observed relationships. The investigation of poor, and rural women in districts of UP with greater marginalization prevent generalization to UP or to other states of India that are culturally different. Further research, including studies with temporal data for households in rural India, are needed to assess determinants of staying members(duration) in programs that integrate health and microfinance.

Policy and program implications

Based on the study findings, policies may aim for suitability of the SHG model to meet the needs of the poorest households in these villages. It is possible that in its current form SHG's are unable to adequately meet the financial requirements of the poorest but are better suited to continue to cater to the poor below the poverty threshold. Efforts are required to communicate the benefit of SHG membership not only to the poor but to all gradients of the poor, especially the poorest, minority religions and marginalized groups(Scheduled Castes/Tribes) . More importantly, there is a need to develop better methods of capturing various gradients of the poor to improve beneficiary selection and targeting in SHG programs.

Second, program planners and implementers can explore different communications strategies to inform potential members of the different reasons for which loans can be availed from SHGs

especially for health reasons. One way that could improve borrowing loans for health reasons is to offer those loans at lower interest rates as compared to loans for enterprise and non-enterprise reasons. In the rural Indian context, the association of limited access to funds, adverse health and deepening poverty is well established. Therefore, encouraging households to take loans for health and illness when required indirectly contributes to SHG functioning overall.

Finally, qualitative research is required to explore reasons why rural poor women may not be joining SHGs in the first place and to inform strategies that support enrolled women to continue membership with a view to improving SHG member enrolment and continued membership overall.

Conclusion:

The SHG model analysed in this paper seeks to target the poorest and most marginalized households in one of India's most populated and developmentally disadvantaged states, Uttar Pradesh. Based on the findings, this study recommends that microfinance programs need to examine their inclusion and retention strategies in favour of poorest household using multidimensional indicators that are better able to capture poverty in its myriad forms. The addition of a maternal health literacy component is found to strengthen the underlying microfinance platform as members gain added value from membership. Both components operating synergistically may also help to interrupt the mutually reinforcing cycle of poverty and poor maternal health. This research also generated evidence on membership profiles of SHGs and provides insights for broader microfinance research elsewhere that seeks to evaluate the outcome of microfinance outcome. By shifting the debate from the effectiveness of microfinance alone without focussing on underlying group composition, we propose the addition of member' profiles and duration of membership as a necessary lens for future microfinance research and practice.

This study does not provide evidence of this.

May review the cost-effectiveness article on health interventions within SHG by Chandrasekhar et al in 2019 on Plos One.

Acknowledgment

We wish to thank Professor Rachel Davey, Health Research Institute(HRI), the University of Canberra for her overall guidance and patiently reviewing the first draft of this paper. The authors would also like to thank the University of Canberra, the Public Health Foundation of

India, and Population Council for supporting inter-organisational research collaboration. Lastly, the authors thank the Bill and Melinda Gates Foundation for supporting the dissemination of this research

References

1. BNP Paribas. Microfinance Barometer 2018: is microfinance profitable? - [Internet]. .[place unknown]:BNP Paribas; 2018 Oct 05 [cited 2019 Oct 28]. Available from: https://group.bnpparibas/en/news/microfinance-barometer-2018-microfinance-profitable
2. Helms B. Access for All Building:Inclusive Financial Access Consultative Group to Assist the Poor [Internet].Washington: World Bank;2006 [cited 2019 Oct 28]. Available from:http://documents.worldbank.org/curated/en/526891468138594047/Access-for-all-building-inclusive-financial-systems
3. Demirguc-Kunt A, Klapper L, Singer D, Ansar S, Hess J. The Global Findex Database 2017:Measuring Financial Inclusion and the Fintech Revolution [Internet]. Washington:World Bank; 2018 [cited 2019 Oct 30]Available from: https://elibrary.worldbank.org/doi/abs/10.1596/978-1-4648-1259-0
4. van Rooyen C, Stewart R, de Wet T. The Impact of Microfinance in Sub-Saharan Africa: A Systematic Review of the Evidence. World Development. 2012;40(11):2249-2262.
5. Bateman M, Chang H-J. Microfinance and the illusion of development: From hubris to nemesis in thirty years. World Econ Rev. 2012;1:13–36.
6. Bateman M. Why Doesn’t Microfinance Work? : the Destructive Rise of Local Neoliberalism. London: Zed Books, 2010.
7. Poletti T, Balabanova D, Ghazaryan O, Kocharyan H, Hakobyan M, Arakelyan K et al. The desirability and feasibility of scaling up community health insurance in low-income settings—Lessons from Armenia. Social Science & Medicine. 2007;64(3):509-520.
8. Milat A, Bauman A, Redman S. Narrative review of models and success factors for

scaling up public health interventions. Implementation Science. 2015;10(1).

9. International Finance Corporation.The World Bank Group and Microfinance [Internet].Washington;WorldBank;[date unknown][cited 2019 Oct 28]. Available from: https://www.ifc.org/wps/wcm/connect/industry_ext_content/ifc_external_corporate_site/financial+institutions/resources/the+world+bank+group+and+microfinance

10. Department MCI. Status Of Microfinance In India 2018-19 [Internet]. Mumbai;Nabard 2018 [cited 2019 Oct 28]Available from: https://www.nabard.org/auth/writereaddata/tender/1207192354SMFI 2018-19.pdf

11. Micro Credit Innovations. Status Of Microfinance In India [Internet].Mumbai;Nabard 2018 [cited 2019 Oct 28]. Available from : https://www.nabard.org/auth/writereaddata/ tender/1207192354SMFI 2018-19.pdf

12. Ruducha J, Hariharan D, Potter J, Ahmad D, Kumar S, Mohanan P et al. Measuring coordination between women's self-help groups and local health systems in rural India: a social network analysis. BMJ Open [Internet]. 2019 [cited 16 January 2020];9(8):e028943. Available from: https://www.ncbi.nlm.nih.gov/pmc/articles/PMC6701569/

13. Mathur P, Agarwal P. Self-help groups: a seed for intrinsic empowerment of Indian rural women. Equality, Diversity and Inclusion: An International Journal. 2017;36(2):182-196.

14. Shah D, Panigrahi S. Determinants of participation of women in self-help groups (SHGs) and credit delivery from formal and informal sources to bpl households in Odisha. Indian Journal of Agriculture Economics 2015;70(3):405–16.

15. Somanathan R, Baland JM, Vandewalle L. Socially disadvantaged groups and microfinance in India. Econ Dev Cult Change. 2019;67(3):537–69.

16. Kumar A.Self-Help Groups , Women's Health and Empowerment: Global Thinking and Contextual Issues Jharkand J Dev Manag Stud. 2006;4(3):2061–79.

17. Emerole CO, Osondu CK, Anyiro CO. Risks, vulnerability and determinants of women farmers' participation in Self-Help-Group (SHG)-led microfinancing in Isuikwuato, Abia State, Nigeria. Sci Pap Ser Manag Econ Eng Agric Rural Dev[Internet]. 2014[cited 2020 Jan 15];14(3):75–81.Available from:http://www.managementjournal . usamv.ro/pdf/vol_14/art11.pdf

18. Borah A. Women empowerment through Self Help Groups-A case study of Barhampur Development Block in Nagaon District of Assam. IOSR J Econ Financ. 2014;4(3):56–62.

19. Pillai T.Self-Help Groups and Women Empowerment (an Empirical Study Done for Mulshi Taluka in Pune District of Maharashtra).[Internet] 2015 [cited 2019 Dec 24];5(3):224–8. Available from: https://www.worldwidejournals.com/indian-journal-of-applied-research-(IJAR)/fileview/March_2015_1492419748__63.pdf

20. Samal S. Self-Help groups and women empowerment : Problems and prospects. Int J Appl Res. 2015;1(13):235–9.

21. National Health MissionVillage Health Nutrition Day (VHND) [Internet]. - Ministry of Health and Family Welfare-Govt of India 2013 [cited 2019 Nov 30]. Available from: http://164.100.154.238/communitisation/village-health-nutrition-day.html

22. Banerjee A, Duflo E, Goldberg N, Karlan D, Osei R, Parienté W, et al. A multifaceted program causes lasting progress for the very poor: Evidence from six countries. Science (80-). 2015;348(6236).

23. International Institute for Population Sciences. National Family Health Survey - 4 2015-16- State Fact Sheet - Uttar Pradesh [Internet] Mumbai. Ministry of Health and Family

Welfare;2016[cited 2019 Oct 20];1–4. Available from : http://rchiips.org/nfhs/pdf/NFHS4/UP_FactSheet.pdf

24. World Bank. India States Briefs – Uttar Pradesh [Internet]. World Bank.2016 [cited 2019 Oct 28]. Available from: https://www.worldbank.org/en/country/india/brief/india-states-briefs-uttar-pradesh

25. Hazra A, Ahmad J,. Khan M.E, Chaturvedi A. Uttar Pradesh Community Mobilization Project: Findings from Scale Up Phase First Cross-Sectional Survey. New Delhi: Population Council India 2015

26. Office of the Registrar General & Census Commissioner, India. Census of India : Provisional Population Totals Paper 1 of 2011: Uttar Pradesh [Internet].[place unknown]Ministry of Home Affairs [cited 2019 Nov 30]. Available from : http://www.censusindia.gov.in/2011-prov-results/prov_data_products_up.html

27. Alkire S, Seth S. Identifying BPL Households. Econ Polit Wkly 2013 Jan 12 2013;XLVIII(2):49–57.

28. Drèze J, Khera R. Recent Social Security Initiatives in India. Vol. 98, World Development. 2017. p. 555–72 .

29. Hilbe J. Modeling Count data.Cambridge: Cambridge University Press;2014

30. Mohanty I, Rammohan A. Child schooling in India: The role of gender. Indian Growth Dev Rev. 2015;8(1):93–108.

31. Gebremedhin TA, Mohanty I. Child schooling in Ethiopia: The role of maternal autonomy. PLoS One. 2016;11(12):1–20.

32. Kinfu Y.Spatial mobility among Indigenous Australians: Patterns and determinants. Canberra: Australian National University. 2005, p. 1-28. (Working Papers in Demography 1975-2006)

33. Vital Statistics Division.2017 SRS Bulletin[Internet] New Delhi, Registrar General, INDIA. 2017 [cited 2019 Nov 22]; Available from : http://www.censusindia.gov.in/vital_statistics/SRS_Bulletins/SRS Bulletin -Sep_2017-Rate-2016.pdf

34. Mohapatra S, Sahoo BK. Determinants of participation in self-help-groups (SHG) and its impact on women empowerment. Indian Growth Dev Rev. 2016;9(1):53–78.

35. Sandhu GK. A Study Of Norms And Functioning Of Self Help Groups (SHGs) In Punjab. J Bus Manag Soc Sci Res [Internet]. 2013 Aug 2[cited 2019 Nov 28];2(8):32–7. Available from : https://pdfs.semanticscholar.org/a9f8/141cc0d8cae4c847dff614f49ac2bf386657.pdf

36. National Informatics Center. Below Poverty Line Certificate Digital India Programme [Internet].New Delhi. Ministry of Electronics & Information Technology- Govt. of India; 2016 Jan 23[cited 2019 Oct 29]. Available from : https://digitalindia.gov.in/content/below-poverty-line-certificate

37. Planning Commission Report of The Expert Group To Review The Methodology For Measurement Of Poverty;New Delhi Government of India. 2014. Available from : http://planningcommission.nic.in/reports/genrep/pov_rep0707.pdf

38. Deendayal Antyodana Yojana, National Rural Livelihoods Mission [Internet].New Delhi: Ministry of Rural Development - Govt. of India 2015 March [cited 2020 Jan 8]. Available from : https://aajeevika.gov.in/en/content/faq

39. Gupta K, Yesudian PP. Evidence of women's empowerment in India: A study of socio-spatial disparities. GeoJournal. 2006;65(4):365–380.

40. Vaessen J, Rivas A, Duvendack M, Jones RP, Leeuw F, van Gils G, et al. The Effects of Microcredit on Women's Control over Household Spending in Developing Countries: A

Systematic Review and Meta-analysis. Campbell Syst Rev. 2014;10(1):1–205.

41. Govil D, Purohit N, Gupta S, Mohanty S. Out-of-pocket expenditure on prenatal and natal care post Janani Suraksha Yojana: a case from Rajasthan, India. Journal of Health, Population and Nutrition. 2016;35(1) . https://www.ncbi.nlm.nih.gov/pmc/articles/PMC5025966/pdf/41043_2016_Article_51.pdf

42. Mohanty S, Srivastava A. Out-of-pocket expenditure on institutional delivery in India. Health Policy and Planning. 2012;28(3):247-262 https://academic.oup.com/heapol/article/28/3/247/551401

43. Kabeer N. Resources, Agency, Achievements: Reflections on the Measurement of Women's Empowerment. Development and Change. 1999;30(3):435-464.

44. Sachar, R Social Economic and Educational Staus of the Muslim Community of India A report.New Delhi: Cabinet Secretariat- Government of India;2006[cited 2020 January 15] Available from: http://www.minorityaffairs.gov.in/sites/default/files/sachar_comm.pdf

45. Fazal T. Millennium Development Goals and Muslims of India.New Delhi Oxfam - India;2013[cited 2020 Jan 16] Available from : https://www.oxfamindia.org/sites/default/files/2018- 10/13%20Millennium%20Development%20Goals%20and%20Muslims%20of%20India.pdf

46. Alam M. Social Exclusion of Muslims in India and Deficient Debates about Affirmative Action. South Asia Research. 2010;30(1):43-65.

47. Demont T, Baland J, Somanathan R. Child Labor and Schooling Decisions among Self-Help Groups Members in Rural India. Economic Development and Cultural Change 2019;33(0):1–51.

48. Khan ME, Agrawal PK, Hazra A, Dixit A, Bhatnagar I, Ahmad J, et al. Maternal and Newborn Health Behaviors in Rural Uttar Pradesh: Findings from Learning Phase[Internet]. New Delhi: Population Council. 2013;[cited 2020 January 15] Available from:https://www.popcouncil.org/uploads/pdfs/2014RH_MaternalNewbornHealthUP.pdf

49. Ministry of Health and Family Welfare India. 10th Common Review Mission.New Delhi: National Health Systems Resource Centre; 2016. Available from: http://nhm.gov.in/images/pdf/monitoring/crm/10th-crm/Report/10th_CRM_Main_Report.pdf

50. 12. Vora K, Koblinsky S, Koblinsky M. Predictors of maternal health services utilization by poor, rural women: a comparative study in Indian States of Gujarat and Tamil Nadu. Journal of Health, Population and Nutrition. 2015;33(1).: http://dx.doi.org/10.1186/s41043-015-0025-x

51. National Rural Livelihoods Mission. Deendayal Antyodaya Yojana -NRLM Financial Inclusion [Internet]. New Delhi: Ministry of Rural Development-Govt.of India 2015 March [cited 2019 Oct 29]. Available from : https://aajeevika.gov.in/content/components/financial-inclusion

52. Ministry of Health & Family Welfare India. A Strategic Approach to Reproductive, Maternal, Newborn, Child and Adolescent Health (RMNCH+A) in India. New Delhi: Govt. of India 2013. Available from : https://nhm.gov.in/images/pdf/RMNCH+A/RMNCH+A_Strategy.pdf

53. Ruducha J, Huang X, Potter J, Hariharan D, Ahmad D, Kumar S, et al. Perceived Social Networks and Newborn Health: Evidence from Self-Help Group Communities in Northern India. Societies. 2018;8(4):92.

Figure Click here to access/download;Figure;Fig 1.tif

Figure 1: Analysis framework: Determinants for SHG membership and Duration for women in Rural Uttar Pradesh, India

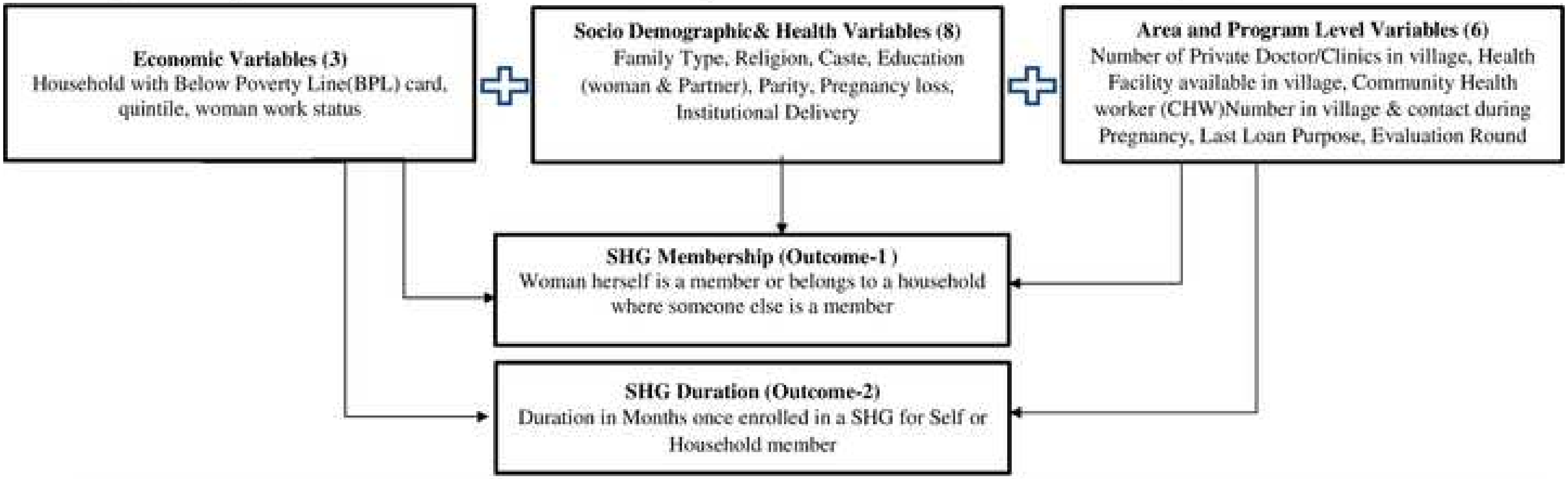

SHG Membership & Duration Model Predictions: :

- Logit (SHG membership) :a(constant) + b1(Household BPL card) + b2 (quintile) + b3(Woman work status) + b4 (family size)+ b5(Family Type) + b6(religion) + b7(caste)+ b8(education woman) + b9(education husband) + b10(parity)+ b11 (Pregnancy loss) + b12(institutional Delivery)+ b13 (num of Pvt Doctors in village) + b14(health facility) + b15(number CHW)+b16 (Contact with CHW)+ b17(Loan purpose)

- Log (SHG Duration):a(constant) + b1(Household BPL card) + b2 (quintile) + b3(Woman work status) + b4 (family size)+ b5(Family Type) + b6(religion) + b7(caste)+ b8(education woman) + b9(education husband) + b10(parity)+ b11 (Pregnancy loss) + b12(institutional Delivery)+ b13 (num of Pvt Doctors in village) + b14(health facility) + b15(number CHW)+b16 (Contact with CHW)+ b17(Loan purpose)

- Interactions included in both models: Household Below Poverty Card # Quintile (Rich to Poor); Household Below Poverty Card # Parity; Survey Round # Loan Purpose; Quintile (Rich to Poor) # Loan Purpose.
