# Supplementary material for: Participation in microfinance based Self Help Groups in India: Who becomes a member and for how long?
Source: PLoS One. 2020 Aug 18;15(8):e0237519. doi: 10.1371/journal.pone.0237519 (PMC7437468; doi:10.1371/journal.pone.0237519)
Supplement: Attachment — Submitted filename: Response to Reviewers.docx [file pone.0237519.s006.docx]

**A point-by-point response letter accompanying the revised manuscript**

We thank the editor and the reviewers for the comments as we think it adds value to the paper and improves the overall presentation. We have also provided a track change version of the manuscript, however, for ease of reference, the page and line numbers indicated in this document refer to the cleaned version of the manuscript

***Reviewers’ Comments to the Authors***

**Reviewer #1:**

Overall the paper is well-developed and well-written. The authors do a commendable job in providing detailed background information on SHGs in India, which help contextualize them with regards to the social and microfinance programs designed to help the poorest households in the country

***Response:*** We thank the reviewer for this comment.

R1-1. *However, the paper includes limited information about the women participants in the SHGs. To provide a better understanding of how the program works, it would be important to know who these women were and what criteria was used to determine their eligibility in the groups.*

***Response:*** We thank the reviewer for this comment. We have now addressed this issue in the revised draft. We have added information detailing the selection criteria used by the program to select an eligible woman for membership in the section titled “project context” on page 8( lines 176-183.)

R1-2. *Given that ‘member selection… is a crucial consideration for the succession of such programs’ (pg. 5), additional information about whether or not these women were from the same community and already knew each other before participation, or whether they were familiar with the SHG program could provide important insights into their engagement with the program.*

***Response:*** We thank the reviewer for this comment. In this study, while eligible women were targeted for membership by the program based on higher levels of marginalization as compared to non-member women; both member and non-members are women from income-poor households living in the same village. Moreover, while the survey collected information from members and non-members in the same village, the data analysis conducted in this paper compares member and non -member across *all* villages.

As SHGs member women are likely to share common characteristics of higher marginalization in a village, it is possible that some women may be familiar with each other before program participation. However, member composition for the SHG is likely to vary with duration of membership depending on how the group function and members repayment ability with non-paying poorer members likely to leave earlier-a theme that is explored in this analysis. Details of member and non-member selection in villages where the program was implemented have been added in the “Project context” section on page 8(lines 176-183.)

R1-3. *The authors conduct a thorough analysis of the important determinants of SHG membership and duration in rural India. Although the survey data is collected from eligible women in both IMFHL program and non-program households (pg. 8), information on what factors were used to determine eligibility for the survey is lacking. It is not clear whether the factors were the same as those used for SHG eligibility, and if so why. Moreover, it is concerning that the paper lacks information on how the control group was created.*

***Response:*** Authors thank the reviewer for pointing this out in the manuscript. We believe addressing this query in the revised draft will improve the overall presentation of the paper. Eligibility details of women in IMFHL program and non-program households are now added in the section titled “Project context” on page 8(lines 176-183)along with details of how control and intervention group were selected on page 8(lines 169-176) by the program for implementation. Moreover, details are also added in the section “ Survey Sampling Approach and Study Population ” page 9 (lines 205-213) to show how control and intervention group were created for the survey for data collection in both rounds.

R1-4. *Although the summary statistics (Table 2) highlight the similarities and differences between the treatment and control groups for some relevant variables, the paper should provide more information about what factors were used to create these groups*

***Response:*** We thank the reviewer for this comment. We have now included details of the program implementation procedure, intervention and how the control and treatment group were implemented on page 8 (lines 169-183), as well as how the survey data were collected in the “Project context” section on page 8 (lines 184-198) and in the “Survey Sampling Approach and Study Population” section on page 9 (lines 205-214). However, it is important to clarify that this paper analyses the determinants of SHG memberships and what determines how long the members continue their membership. In this process, while the dataset used in this paper also contains information on IMFHL program intervention, control and treatment group selection, our research question is concerned with determinants of SHG membership and duration only.

R1-5. *I am also concerned about the potential for multicollinearity issues in the model due to the inclusion of variables that might be correlated with each other. For example, the variable working status of eligible women for cash, kind, or both, a proxy for household poverty, could be correlated to women’s education levels. If women with higher education were more likely to be working relative to their less-educated counterparts, this may bias their impact on SHG membership and duration. Additionally, if households with educated partners were less likely to be poor relative to those with lower levels of education, it would differently impact their participation in the program, would be interesting to see how interactions between the education levels and other relevant variables might impact the results of the study. Some robustness checks would be useful to ensure the results are not biased due to multicollinearity.*

***Response:*** We thank the reviewer for this comment and have provided an explanation here. In the original analysis, we were interested in exploring the socio-economic determinants that may be operating in a rural income poor population to influence membership. However, the predominant occupation of women in poor rural settings in Uttar Pradesh, India tends to be in the informal agricultural sector, which is more easily accessible to women as part of their households in villages. Additionally, we also see from the summary statistics in our study that only a minority of women 13 % (non-SHG) & 17% (SHG) are working. Furthermore, in a rural setting where most women are income poor, we find that working status is less likely to be correlated with education. In such a setting, the likelihood that educated or women working are SHG members are more likely to be due to intrinsic factors or determinants linked to the women such as individual self -agency or empowerment or the extent of her social/peer networks that introduce her to SHGs. Hence, the correlation between education and working status in such settings is weak. We moreover have assessed the likelihood of multicollinearity among different variables in our analysis and have conducted robustness checks that are also explained here. Firstly, we examined the correlation of the estimated covariates in this analysis across each explanatory variable using a correlation matrix and found that the correlations are low. As the majority of our explanatory variables were categorical in nature and comprised dummy variable, standard post estimation tests would not be appropriate. We, therefore, used the polychoric correlation test to generate a matrix that revealed low (less than 0.25) correlation in our variables, and we can, therefore, conclude that MC is not an issue for the variables in the analysis

R1-6 *Additionally, if households with educated partners were less likely to be poor relative to those with lower levels of education, it would differently impact their participation in the program, would be interesting to see how interactions between the education levels and other relevant variables might impact the results of the study. Some robustness checks would be useful to ensure the results are not biased due to multicollinearity.*

**Response:** We thank the reviewer and agree with their concern that there may potentially be multicollinearity issues between education and other socio-economic variables in the model. The majority(66%) of women in our sample have received some form of education, and that influences the results. Moreover, the summary statistics reveal that majority of women in both SHG groups (67% or 5403 women/8048) and Non-SHG groups (68% or 4755 women/7252) have some level of education. Therefore, the regression results in the full SHG membership and duration model are predominantly driven by the effect of women with some level of education compared to those with no schooling/education. We also separately conducted the following robustness check for examining if we need to interact education with other socio-economic variables and if there is multicollinearity issue in the model for SHG program membership.

We separated the full study sample comprising of 15,300 women on the basis of eligible woman’s education as ‘no education’ and ‘any education’ and re-ran the full regression model for both Membership and Duration independently for both groups. We assessed the change in coefficients in both groups (no education and any education) against the full model reported in the paper. We found that the regression coefficients in the full model in the paper are almost similar to the coefficients of the models we separately ran for the educated group.

Moreover, we also assessed the mean duration of membership among educated and uneducated women and found only a four-month difference between them (Mean membership duration in months for women with no education=19 months; SD: 28 versus mean membership duration for women with any education=23 months: SD: 32 months). Additionally, we conducted a non-parametric test (Wilcoxon rank-sum test) to assess if there is a significant difference in the duration of SHG membership between the women with some level of education and those who have no education which there was not. Thus, substantiating that we do not need to interact with the education variable with other socio-economic variables in the regressions for SHG duration.

R1-7. *Furthermore, the paper would benefit from a detailed discussion of some of its results. An interesting finding is that, accounting only for loan purposes, when women borrowed funds for health reasons they were less likely to become SHG members but more likely to continue their membership for a longer period. However, when asset-based wealth quintiles were included in the analysis, the results suggested that women in the lower wealth quintiles were most likely to become SHG members when they borrowed for health reasons, but would likely continue the membership for longer if they borrowed for income-generating activities only. The paper does not include enough discussion and clarity on this seemingly contradicting results on the impact of loan purpose on membership and duration*

***Response:*** ***:*** We thank the reviewer for this comment. We have now revised and re-run our regression models, including a more refined wealth index and the revised results are presented in the revised manuscript. Correspondingly the results and discussion have also been revised in the revised manuscript in the following sections: i.“Results” page 18 (lines 369-382), pg 20 (lines 411-434) and page 21(lines 441-446); ii.“Discussion” page29 lines 511-550”; iii.“Targeting the Poorest from the Lowest Wealth Quintile Households” page 32-33 (lines 577-601) and lastly, iv. “Effect of Borrowing on SHG Membership and Duration” page 33-35 (lines 619-663).

The revised results show that poor households captured by BPL indicator *are more likely to* be SHG members than non-BPL cardholders. Moreover, the inclusion of wealth quintile shows that overall the odds of membership *increases* as the wealth quintiles *descends* from marginally poor households to the poorest (quintile 5) in this sample suggesting that all categories of poor are more likely to be captured by the program for membership. In fact, the poorest households are almost 70 per cent or 1.71 times *more likely* to be members as compared to marginally poor households in the same villages. However, the duration analysis shows that once enrolled into SHGs, women belonging to households with below poverty line (BPL) cards *are less likely* to remain, members, as compared to households without BPL cards (IRR=0.99, 95% CI:0.91-1.07, p>0.05). The likelihood of staying members (duration) *decreases* across the poverty gradient represented by the wealth quintile with the poorest (quintile5) households being 0.78 times *less* likely to stay members as compared to the marginally poor (IRR=0.78, 95%CI:0.71-0.85, p<0.01). The aim of the paper and discussion highlights the importance of using wealth quintile as an indicator to track the movement of the poorer households in SHG (membership) and exit(through duration analysis).

Similarly, we have revised the results and discussion in this paper for the impact of loan borrowing on SHG membership and duration that shows when women borrowed funds for any purpose, they are more likely to become SHG members and are also more likely to continue membership for a longer period as is originally envisaged by microfinance advocates. Encouragingly, households that borrowed funds for health reasons such as delivery expense or treatment of illness were 1.71 times more likely to become SHG members and 1.38 times more likely to stay members which may reflect their ability to gain financial access from SHG membership and be able to pay back loans. The results show that poorer households in all quintiles( 2,3, 4 and 5) in contrast to marginally poor households(quintile 1), are less likely to *become SHG* *members* when they borrowed for health-related expenses. These findings is supported by the claim made in the paper that poor households have reduced capacity to pay back borrowed loans when they borrow for illness as they are less likely to be able to use these funds for income generation.

R1-8. *Similarly, the results show that women belonging to Scheduled Castes were more likely to be members of the program compared to those from the majority Hindu general caste. Given that the caste system is highly prevalent in the Indian society, it would be interesting if the authors could discuss this relationship in the context of SHGs and its implications on credit accessibility to socially marginalized groups.*

***Response:*** Authors thank the reviewer for this insightful comment. Details of the caste relationship in the context of SHG membership, duration and implication on credit utilization have been added in the revised manuscript, section “Impact of Social class (caste) on SHG Membership and Duration” on page 38 (lines 725-735). However, it is important to highlight that , the IHLMF program targeted disadvantaged households such as those belonging to scheduled caste that typically experience higher poverty and social marginalization in UP. The literature also finds that because scheduled caste households are more likely to be socially ostracized with limited credit access, SHGs programs in India like the IHMFL program should focus on scheduled caste households to improve their access to resources. Membership in SHGs is seen as an approach not only to improve credit access but also as an empowering tool for women belonging to SC households to build self-confidence and individual agency to overcome restrictive social norms.

R1-9. *The findings of the study provide important insights into factors that determine participation in SHG programs and the duration of membership. However, the results may need to be presented in a clear way for readers to better understand the implications of this study. Details regarding the suitability of the various count data models and Table 1 (pg. 13) could be included in the appendix.*

***Response:*** We thank the reviewer for this comment and agree with them. Presentation of results is now simplified by providing only results of the full models for SHG membership and duration in table 3. As suggested, the details of the count data model fit(Table 1)page 12 (in the original draft)have now been included as a table in the S2 appendix

R1-10*. Summary statistics presented in Table 2 (pg. 14-16) is highly informative. However, a shorter version of the table would suffice to highlight the key similarities and differences between the treatment and control groups. Moreover, Table 3 in its current form is too long and difficult to follow. I recommend that the authors include a shorter version of the table such that it highlights the main results. More detailed versions of Tables 2 and 3 can be included in the appendix*

***Response:*** We thank the reviewer for these comments and suggestions. We have now revised the manuscript based on this comment and believe this revision improved the clarity and the presentation of our paper. As suggested, table 2 and 3, have been shortened in the paper with full versions included as table(s) in the S3 and S4 appendix respectively. Importantly, table 3 is shortened to provide only final regression models for SHG membership and duration. Additional results from SHG membership regression models(I and II) are presented in a table 3a provided in the appendix.

**Reviewer #2:**

Well written paper on an important topic. Suggest few minor changes, most important being in the conclusion. Have attached the manuscript with my comments for your consideration. The tables also needs some review.

***Response:*** We thank the reviewer for these comments and have addressed comments in the paper as follows.

**Method:**

R2-1. *P. 13, Line 361:”* Firstly, to assess the determinants of SHG membership, three forward logit regression models were performed” **Reviewer Comment** -“*Is this necessary, makes it very confusing for reader, without much added value”*

***Response*** We thank the reviewer for this suggestion which was also raised by reviewer one. We have revised the models presented in the paper to only present the final regression model results for SHG membership in table 3 (pg 23,line 486) whereas the additional models I and II are provided in the table in S4appendix. Accordingly, the results section(pg 18-23) and discussion section(pg 28-44) now also elaborate on the final model in detail.

**Summary statistics**

R2-2. *P. 16, Lines 517-82: Some info on what % of households are part of SHGs in the 20 districts you sampled would be useful. This will give an understanding of the spread of SHGs in the community*

***Response:*** We thank the reviewer for these comments. Details of SHG household coverage are included in the summary statistics and show that 53 per cent of households across districts were SHG members representing almost 8109 women. Please check the revised manuscript on page 15(line 351) table 2.

R2-3. “However, a larger number of non-SHG (15%) households as compared to SHG (13%) members” **Reviewer Comment-** *15% not much higher than 13%*

***Response:*** We thank the reviewer for this comment. We have edited the statement in the revised manuscript on page 15 line 348-350 to now read as “…an almost similar proportion of non-SHG (15%) households as compared to SHG (13%) members…”

***Minor comments:***

R2-4. *P. 3, Table2 has some errors, have highlighted these, also may be useful to just have descriptive of independent variables*

***Response:*** We thank the reviewer for highlighting the error. Table 2 on page 15-16 has been corrected in the revised manuscript. Moreover, summary statistics in table 2 has also been shortened as suggested by both reviewers and the detailed table enclosed in S3 appendix

R2-5. *P. 3, Table 2* **Eligible Woman (EW)Age in completed year: Mean age=26**

**Reviewer Comment** *-Is the mean age the norm for all SHGs, or is this result of your sampling?*

***Response:*** This is the mean age of survey respondents in the study sample only. The survey sampled SHG and non-SHG member who had recently delivered an infant in the last 12 months preceding the survey, which explains the relatively younger age range seen here. As per the microfinance literature from India, however, the age of most SHG members is more likely to be older women who have completed their reproductive life cycle (18-45) or are in the upper segment of the reproductive life cycle.

R2-6. *Pg 24 Table 3 May be really useful for authors to pick model 3 to report in paper and refer to other two models if required in discussion, rather than present all three models in results and in discussion This may significantly improve clarity of results and discussion section. Also may be useful to carefully consider the value of all the variables in model 3.*

***Response:*** We agree with the feedback provided by both reviewers regarding table 3 and the presentation of the results. Accordingly, table 3 has been shortened in the revised draft to only provide results of the final regression model for SHG membership(model III) and duration with detailed models provided in the appendix. The results section (pg18-23) and discussion section(pg 28-44) in the revised draft has similarly been revised to only present model 3 findings of SHG membership to improve clarity.

**Discussion**

*R2-7. P 31 Line 530: Some discussion on the practicality of using wealth quintiles for government programs would be useful. It is an easy method to use rather generally used for analyzing a program in a study, but tough to be used as a method for identifying poor in national programs.*

***Response:*** We thank the reviewer for the comment. The paper has included additional lines 547-550 on pages 30-31 in the revised draft to clarify the benefits of using BPL card in programs.

R2-8. *P. 46, Line688:* “While SHGs usually comprise of older women that may have completed their families” **Reviewer Comment-** *Please refer to my question re mean age.*

***Response:*** The question has been addressed in response to reviewer question 5 above.

**Discussion**

R2-9. *P. 47, Lines 711-716: “*Therefore, adding a health intervention to a microfinance program seems to strengthen the model overall by encouraging membership, and may offer evidence in favour of similar integrated programs being scaled up..” ” **Reviewer Comment-** *Unclear if this study provides any such evidence re benefits of adding health intervention to SHGs. Suggesting that such integrated programs should be scaled up requires much clear evidence and information on cost effect,so maybe best to avoid this here*

***Response:*** We thank the reviewer for this comment. The inference has been edited and rephrased in the revised manuscript on page 40( lines 780-784) to say the following: ”While additional research is still required, our findings show the effects of layering health on microfinance, and provides evidence to policymakers/advocates keen on adding health intervention to India’s federal poverty alleviation plan the N.R.LM which currently reaches 620 million households and present an opportunity to accelerate towards the 2030 Sustainable Development Goals for health and poverty reduction”

**Conclusion**

R2-10. Pg 50 lines 803-806: “The addition of a maternal health literacy component is found to strengthen the underlying microfinance platform as members gain added value from membership. Both components operating synergistically may also help to interrupt the mutually reinforcing cycle of poverty and poor maternal health”. **Reviewer Comment- *“****this study does not provide evidence of this.”* *May review the cost-effectiveness article on health interventions within SHG by Chandrasekhar et al. in 2019 on Plos One.*

***Response:*** We agree and thank the reviewer for this comment. We have revised the conclusion in the revised manuscript on page 44 Lines 872-876 accordingly and have also incorporated findings from another state of India (Bihar) from the *suggested study* in these lines.
