# Supplementary material for: Participation in microfinance based Self Help Groups in India: Who becomes a member and for how long?
Source: PLoS One. 2020 Aug 18;15(8):e0237519. doi: 10.1371/journal.pone.0237519 (PMC7437468; doi:10.1371/journal.pone.0237519)
Supplement: Attachment — Submitted filename: Response to Reviewers.docx [file pone.0237519.s007.docx]

**A point-by-point response letter accompanying the manuscript’s second revision**

We thank the editor and the reviewers for the additional comments that we have addressed in the second revision of the paper (Jul 21 2020). Along with the letter, the authors are also submitting a clean version of the manuscript and a track changed version with comments addressed.

***Reviewers’ Comments to the Authors***

**Reviewer #3:**

‘Very pertinently, Authors have taken care of reviewers comments step by step and finally, the outcome in the form of paper (revised version) is in good shape. But there are certain issues which I would like to point out as following:

‘186 development regions of Uttar Pradesh (55). Under the IHLMF program, is wrongly put, it should be (IMFHL) Integrated Microfinance and Health Literacy 190 plus health (intervention blocks) in the IHLMF program.is wrongly put and should be (IMFHL) Integrated Microfinance and Health Literacy 215 (out of a total of 80 districts in U.P state). U.P. is administratively subdivided into 80 districts is wrong as Uttar Pradesh is comprised of 75 districts. (Uttar Pradesh is divided into 75 Districts and 18 Divisions.Mar 2, 2020 Uttar Pradesh District Map, List of Districts in Uttar Pradesh) [www.mapsofindia.com](https://protect-au.mimecast.com/s/0N0lCgZoAYslnMKMcoFF4t?domain=mapsofindia.com)

If these corrections are inserted I think the paper has touched upon a very important issue of poverty eradication through SHG policy in the State of Uttar Pradesh.
I strongly recommends the publication of the paper.’

***Response:*** We thank the reviewer for these comments and suggested corrections. We appreciate the reviewer’s recommendation for the publication of the paper.

R3-1.” 186-*Under the IHLMF program, is wrongly put, it should be (IMFHL) Integrated Microfinance and Health Literacy*

*190 plus health (intervention blocks) in the IHLMF program.is wrongly put and should be (IMFHL) Integrated Microfinance and Health Literacy.’*

***Response:*** We thank the reviewer for this comment. We have now revised the abbreviation IHLMF to appear as IMFHL in the revised draft. Changes were made in the following pages and line numbers where the abbreviation now appears as **IMFHL**: page 8 (line number 169 and 173); page 9 (line number 214); page 20 (line number 423); page 22 (line number 470);page 38 (line number 730 and 737); page 39 (line number 767 and 768).

R3-2. ‘*215 (out of a total of 80 districts in U.P state). U.P. is administratively subdivided into 80 districts is wrong as Uttar Pradesh is comprised of 75 districts. (Uttar Pradesh is divided into 75 Districts and 18 Divisions. Mar 2, 2020 Uttar Pradesh District Map, List of Districts in Uttar Pradesh)* [*www.mapsofindia.com*](https://protect-au.mimecast.com/s/0N0lCgZoAYslnMKMcoFF4t?domain=mapsofindia.com)*.’*

***Response:*** We thank the reviewer for this comment. We have revised the number of districts in Uttar Pradesh from 80 to **75** in the revised manuscript on page 9 (line 200).
