# Supplementary material for: Participation in microfinance based Self Help Groups in India: Who becomes a member and for how long?
Source: PLoS One. 2020 Aug 18;15(8):e0237519. doi: 10.1371/journal.pone.0237519 (PMC7437468; doi:10.1371/journal.pone.0237519)
Supplement: Attachment — Submitted filename: Response to Reviewers.docx [file pone.0237519.s008.docx]

**A point-by-point response letter accompanying the manuscript’s second revision**

We thank the editor for the additional comments that we have addressed in the third revision of the paper (Jul 24th 2020). Along with the letter, the authors are also submitting a clean version of the manuscript and a track changed version with comments addressed.

***Reviewers’ Comments to the Editor***

**Editor’s(E) Overall Comment :**

‘No doubt your paper is making significant contributions and it will be recommended for publication on behalf of me for sure. However, can you consider the following minor comments from my end?

***E-1:*** *“Can you check the reference citation carefully? It should be chronological in order. The first cited reference should be first. Why reference no. 55 is coming much ahead of its order.”*

***Response:*** We thank the editor for highlighting this and the suggested corrections. We have revised the paper to ensure that references are in chronological order.

E-1 *“Overall studies from India found that vulnerable households at the threshold of poverty get pulled deeper into poverty when exposed to maternal and newborn health issues due to lack of affordable financial sources to pay for treatment that is mainly Out of Pocket (41, 42).”. Does any of the following papers assess newborn health issues and OOPE? One is focused on institutional deliveries and the other is on prenatal and natal care. Both suffer from significant data and methodological limitations. Moreover, none of them are in the Uttar Pradesh context. Considering health systems are vary considerably across the states, OOPE and Catastrophic OOPE also vary across the states.
[41] Govil D, Purohit N, Gupta S, Mohanty S. Out-of-pocket expenditure on prenatal and
natal care post-Janani Suraksha Yojana: a case from Rajasthan, India. Journal of Health,
Population and Nutrition. 2016; 35(1).
[42] Mohanty S, Srivastava A. Out-of-pocket expenditure on institutional delivery in India. Health Policy and Planning. 2012; 28(3):247-262 1010* [*https://academic.oup.com/heapol/article/28/3/247/551401*](https://academic.oup.com/heapol/article/28/3/247/551401)
***Response:***  We thank the editor for guiding us to additional references which have been included in the paper(see next response). The literature makes an attribution for higher maternal costs during delivery/intrapartum events to be also closely linked to newborn health expenses. However, we have added another reference from Uttar Pradesh on page 35, reference number 39 to highlight high OOPE associated with neonatal illness for the poor.

**E-2** *Thus, try to cite more relevant literature for the sentence that your writing. Why do you ignore other more comprehensive studies which considered greater details of background characteristics and also assessed postnatal care too? I have given some references as below which overcome the several data and methodological limitations compared to the studies you have cited. You can refer to them if you would like to.*

- *Goli, S., Rammohan, A., and Pradhan, J., 2016. High spending on maternity care in India: What are the factors explaining it?. PloS one, 11(6), p.e0156437.*
- *Goli, S., and Rammohan, A., 2018. Out-of-pocket expenditure on maternity care for hospital births in Uttar Pradesh, India. Health economics review, 8(1), p.5. These are just an example, but you need to check other references too..’*

***Response:*** We thank the editor for sharing these resources. The references shared add value to the claims made in the paper and we are thankful to the editor for highlighting these articles. Previously, these articles had not come up in early search result on google scholar due to the combination of search words used. The timely sharing of these resources will also be useful in guiding and inclusion in our other papers currently under development. The suggested articles have been included in page 35(lines 691) Along with useful insights included in lines 692-693 from the suggested references.

**E-3**. ‘*I am giving one more reference which is a recent comprehensive study that documents on “Poverty, Employment, Health and Education, and Access to various Social Safety nets” across Six Social Religious Groups. You can refer to it if you would like to.
Kumar S, Prashan F, Trivedi K, Goli S. BACKWARD AND DALIT MUSLIMS: Education, Employment and Poverty..’*

***Response:*** We thank the editor for sharing the reference to this book. It will be a useful resource for our future resource, which will also be facilitated by greater access to book copies.
